# Genetic dissection of root traits in barley identifies major QTLs and domestication signature

**DOI:** 10.1007/s00299-026-03852-3

**Published:** 2026-05-23

**Authors:** Giuseppe Sangiorgi, Cristian Forestan, Francesco Camerlengo, Giuseppe Sciara, Matteo Bozzoli, Agostino Fricano, Alessandro Tondelli, Riccardo Fusi, Rahul Bhosale, Roberto Tuberosa, Silvio Salvi

**Affiliations:** 1 Department of Agricultural and Food Sciences (DISTAL), University of Bologna, Viale Fanin 44, 40127 Bologna, Italy; 2 CREA – Research Centre for Genomics and Bioinformatics, Via San Protaso 69, 29017 Fiorenzuola d’Arda, Piacenza Italy; 3 School of Biosciences, University of Nottingham, Sutton Bonington Campus, Nottingham, LE12 5RD UK; 4 Present Address: Leibniz Institute of Plant Genetics & Crop Plant Research (IPK), OT Gatersleben, Corrensstr 3, 06466 Seeland, Germany

**Keywords:** Barley, Domestication, GWAS, QTL, Root architecture

## Abstract

**Key message:**

Using genome-wide association analysis in a barley germplasm panel, we identified root trait QTLs, domestication-driven morphological shifts, and candidate genes to guide breeding for improved root ideotypes in modern cultivars.

**Abstract:**

Root system architecture (RSA) determines the plant’s ability to anchor to soil and absorb water and nutrients, thereby affecting productivity and stress tolerance. In this study, we investigated phenotypic variation for seven root traits at the seedling stage, performed a genome-wide association study (GWAS), and identified novel quantitative trait loci (QTLs) and candidate genes using a germplasm collection representing global barley diversity. Traits heritability ranged from 0.70 to 0.88. Domestication and breeding syndromes were detected for seminal root number (SRN), which increased from 4.2 roots in wild accessions to 5.9 in landraces to 6.3 in modern cultivars. Similarly, root growth angle (RGA) widened from 42.9° in wild accessions to 60.3° in modern cultivars. Lateral root density and length increased from wild barley to cultivars, while average seminal root length (ARL) shortened. GWAS identified 106 QTLs explaining 25–61% of the phenotypic variation per trait. Candidate genes at GWAS peaks for SRN encoded *ARABIDILLO*- and *WD-40*-related proteins, an exocyst complex component, and a serine protease; UDP-glycosyltransferase for RGA; and jasmonate receptor and F-box encoding genes for ARL. Our findings provide valuable insights for molecular biologists and breeders to design and implement root architecture ideotypes in new cultivars, enhancing their adaptability to challenging environments.

**Supplementary Information:**

The online version contains supplementary material available at 10.1007/s00299-026-03852-3.

## Introduction

Root system architecture (RSA) determines the volume of the soil explored, the efficiency of water and mineral nutrient uptake and a plant’s capability to withstand drought, nutrient deficiency, and soil compaction—challenges expected to worsen in the near future (Voss-Fels et al. 2018b; Bailey-Serres et al. 2019; Ober et al. 2021; Rezaei et al. 2023). Empirical studies have established correlations between RSA variation and tolerance to abiotic stresses (Uga et al. 2013; Chen et al. 2014; Saengwilai et al. 2014; Robinson et al. 2018) and these relationships were further explored in recent reviews (Maurel and Nacry 2020; Siddiqui et al. 2021; Lynch 2022; Maqbool et al. 2022; Kalra et al. 2024; van der Bom et al. 2025). However, a deeper understanding of the molecular genetic control of RSA development and its variation within species is required to support breeding programs aimed at modifying RSA for improving crop stress resilience and sustainability (Maurel and Nacry 2020; Maqbool et al. 2022). Despite the recognized importance of root system architecture, the genetic basis of many RSA traits and their evolution during crop domestication and modern breeding remain poorly understood.

Due to the complexity and cumbersome nature of root phenotyping, molecular genetic studies and breeding programs have historically overlooked root studies (McGrail et al. 2020; van der Bom et al. 2020). However, this has changed in recent years with the development of new procedures and protocols that have made root phenotyping more feasible (Atkinson et al. 2019; Watt et al. 2020). Many of these efforts have focused on young seedlings under controlled conditions. Although such systems cannot fully capture the complexity of root system architecture in field-grown plants, they enable the efficient screening of large germplasm collections and facilitate the identification of genetic loci controlling early root traits that may influence plant establishment and resource acquisition (van der Bom et al. 2020). The extent to which seedling traits reflect RSA at later developmental stages remains controversial. While in some cases the correlation between controlled and field conditions for similar traits was low (e.g., Cabeza), in other cases it was sizeable (Ali et al. 2015; Maccaferri et al. 2016).

Barley (*Hordeum vulgare* L.) is the fourth most important cereal in the world after wheat, rice, and maize. Its area of distribution and cultivation is very large, suggesting a great ability to adapt to different environmental conditions (Russell et al. 2016). Barley is also a model species within cultivated Poaceae thanks to its fully diploid genome (unlike polyploid wheat or oats) and the well-known interspecific synteny relationships, which facilitate the characterization of genes and mutants and the transfer of molecular genetic information to phylogenetically related species (Rossini et al. 2018). Like other monocots, barley develops both an embryonic and a post-embryonic root system (Rossini et al. 2018). The embryonic root system is usually composed of three to eight seminal roots, including the primary root, while the post-embryonic system comprises nodal roots arising from the basal stem nodes. Both seminal and nodal roots produce lateral roots and root hairs, which together form the plant’s main interface with the soil. Seminal roots play a crucial role in early seedling establishment and remain active throughout the plant’s life. Under severe water deprivation after germination, seminal roots may be the only type of roots that develop, as nodal root growth can be strongly prevented (Sebastian et al. 2016). In barley, variation for RSA traits was mostly shown to be under quantitative genetic control. QTLs for seminal root number, growth angle, dry weight, and other traits have been mapped in both experimental populations and germplasm collections (Chloupek et al. 2006; Ahmad Naz et al. 2012; Arifuzzaman et al. 2014; Naz et al. 2014; Robinson et al. 2016, 2018; Reinert et al. 2016; Jia et al. 2019; Abdel-Ghani et al. 2019; Cabeza et al. 2025). To date, only a few mutants (Bovina et al. 2011) have been described, and Mendelian genes associated with barley RSA include *VRN1* (Asp et al. 2011; Voss-Fels et al. 2018a), *HvDRO1* and *HvSOR1* (Nakano et al. 2022), *Enhanced Gravitropism-1* (*EGT1*), and *Enhanced Gravitropism 2* (*EGT2)*, which affect root growth angle and gravitropism (Kirschner et al. 2021; Fusi et al., 2022), and *HvPin1a*, which affects root length and anatomy (Fusi et al. 2024). Nevertheless, the number of cloned genes underlying natural variation for RSA traits in barley remains limited, highlighting the need for further genetic dissection using diverse germplasm collections.

Despite their importance, fewer studies have addressed the shift of RSA in relation to domestication and breeding compared to shoot traits (Isaac et al. 2021; Alam and Purugganan 2024). A recent study involving Australian landraces and cultivars suggests that commercial breeding may have inadvertently influenced root system architecture (RSA) through the selection of correlated polygenic traits (Aldiss et al. 2025). Barley, maize, and emmer wheat, when compared to their wild ancestors, have been shown to carry a higher number of seminal roots, a trait likely selected to enhance nutrient uptake and support seedling establishment (reviewed in Alam). Identifying clear trends for other root traits has been more challenging, likely due to the influence of confounding factors and trade-offs, including species pre-adaptation, crop management practices, and root–microbe interactions. In maize, for example, comparisons across historical cultivar series have revealed a reduction in root growth angle (i.e., steeper roots) and an increase in the number of seminal roots (Ren et al. 2022; Yu et al. 2024). However, comparable analyses across globally diverse barley germplasm collections remain limited, and the extent to which domestication and breeding have shaped RSA traits in barley is still unclear.

In order to address knowledge gaps highlighted above, in this study we investigated RSA variation at the seedling stage in a barley germplasm collection that represents the global diversity and performed GWAS for RSA traits using exome-based SNP markers. Based on these analyses, we explored the evolutionary trajectories of these traits in barley germplasm including signatures of domestication and selection and identified novel QTLs and candidate genes that are relevant for root development. This work provides new insights into the genetic basis of RSA in barley, offering novel avenues to guide the development of root traits for improved crop resilience and productivity in challenging environments.

## Materials and methods

### Plant material

The almost complete barley germplasm collection of the WHEAt and barley Legacy for Breeding Improvement—WHEALBI (Bustos-Korts et al. 2019; Sow et al. 2025) project (EU FP7 no. FP7-613,556) was utilized in this study. The WHEALBI barley collection includes wild accessions, landraces and cultivars, both two-rowed and six-rowed, originating from four main different geographical areas, namely Asia, Africa, Europe, and the Middle East. We grew 476 accessions and successfully obtained root phenotypic data for 448 accessions which were used for biometrical analysis. Genotypic data were available for 459 accessions (Bustos-Korts et al. 2019). Eventually, complete phenotypic and genotypic data were available for 400 accessions, which were used for GWAS.

### Growth conditions

For each accession, ten representative seeds were selected and treated by immersion in a 10% NaClO solution for 7 min and rinsed first with tap water and finally with ddH_2_O. Seeds were pre-germinated on blotting paper in Petri dishes for 24 h at 28°C. A 50 × 50 cm sheet of blotting paper (the same used for pre-germination), soaked in deionized water, was adhered to a black polycarbonate panel (38.5 × 42.5 cm). On this paper sheet, two horizontal lines were drawn at 3.0 and 5.5 cm from the upper edge, with a pencil. Five germinated seeds, chosen in such a way that they were as homogeneous as possible in terms of size and state of germination, were placed on the paper sheet in correspondence with the upper line, with the radicle facing downward and the ventral furrow facing the sheet. Subsequently, a second sheet of paper soaked in deionized water was placed over the first. Panels (from 20 to 27) were placed vertically in tanks containing 10 L of ddH_2_O, the level (5 cm) of which was kept constant throughout the growth period. An additional polycarbonate panel was placed in front of the first to prevent light from reaching the roots of the plants in the first panel. The tanks were placed in a growth chamber with a photoperiod of 16–8 h (light–darkness) and a temperature of 22°C and were kept for 14 days. Two consecutive replicates were performed, considering a single panel containing five seedling plants as one replicate.

In order to validate the results obtained in the panel-based semi-hydroponic system, accessions with the most extreme (maximum and minimum) phenotypic value for each of the following traits RGA (accessions min: WB-075 and WB-363; max: WB-146 and WB-147), LRD/LRL (min: WB-075, WB-415 and WB-480; max: WB-240, WB-254 and WB-311) and SRN (min: WB-494 and WB-497; max: WB-240 and WB-384) were grown and analyzed using a soil-filled rhizotron system. A rhizotron consisted of a box assembled using a black polycarbonate panel (34 × 44 cm), a glass plate of the same dimension, held together by four metal clips, and with lateral shims leaving a gap of 0.5 cm. The empty volume was filled with compacted universal soil (BR light professional mix, Vigorplant). Two pre-germinated seeds were placed 2 cm below the upper edge, equidistant from the lateral sides. The experiment was set as a completely randomized experiment including 3 rhizotrons (2 plants per rhizotron) per barley line, for a total of 52 rhizotrons (104 plants). The rhizotrons were positioned at a 60° inclination in a plastic rack appropriately built, with the glass facing downward on a dark support and maintained in a greenhouse under controlled conditions (22°C/16 h day, 18°C/8 h night) with natural light supplemented by 400 W high-pressure sodium lamps (Sylvania SHP-TS 400W Grolux). Plants were watered every 3 days and grown for 14 days. Root system images were acquired at 14 days using an Epson Expression 12000XL scanner, processed with Epson Scan 2 software, and analyzed with ImageJ to measure seminal root angles. The number of seminal roots was counted manually, whereas the lateral roots were traced with one-pixel-width trace using GIMP 2.10.38. Lateral root number was determined as the number of traced lines, whereas lateral root length was determined as the total number of pixels (provided in GIMPS) divided by the number of lateral roots.

### Root system phenotyping

At day 14, each panel was digitally photographed using a Nikon D5600 camera, positioned 60 cm from the panel, using DigiCam Control (http://digicamcontrol.com/). The images were saved in jpg format and subsequently analyzed with ImageJ (http://imageJ.nih.gov/ij/) for the root growth angles calculation, and Gimp software (https://www.gimp.org) for extraction of total root length (further details are provided in Table S1). Seven phenotypic traits were finally collected (Table 1 and Table S2). Additionally, for each seedling, the number of seminal roots was counted, and root dry weight was measured after drying in an oven at 68°C for 5 days. For lateral root density, a scoring system ranging from 1 to 4 (1 = < 4 lateral roots per seminal root, 4 = > 12 lateral roots per seminal root) was applied. For lateral root length, accessions were scored as follows: 0 = no lateral roots; 1 = lateral roots up to 6 mm; 2 = lateral roots > 6 mm, considering the portion of axial roots (both primary and seminal roots) between 3 and 15 cm from the seed (details of the phenotypic data collection are provided in Table S1 and images used as reference are provided in Fig. S1). Thousand kernel weight (TKW) was collected and used for correlation with root architectural traits in order to rule out potential maternal effects. All phenotypic data and lines information are provided in Table S2.

**Table 1:** Phenotypic variation and heritability of seminal root traits collected in this study

Acronym	Trait	Unit	Mean (± SD)	Range	CV (%)	Heritability
ARL	Average root length	cm	26.51 ± 2.70	18.51–34.72	10.2	0.71
LRD	Lateral root density	score	2.74 ± 0.71	1.09–4.04	26.0	0.81
LRL	Lateral root length	score	1.20 ± 0.41	0.11–2.00	34.3	0.74
RDW	Root dry weight	mg	17.81 ± 3.36	8.86–28.01	18.9	0.70
RGA	Root growth angle	degree	55.88 ± 14.77	16.39–118.77	26.4	0.78
RTL	Total root length	cm	155.43 ± 14.52	88.97–230.50	14.8	0.78
SRN	Number of seminal roots	number	5.91 ± 0.79	3.29–8.20	13.5	0.88
TKW	Thousand kernel weight	g	51.51 ± 7.92	27.00–74.00	15.3	–

Distributions of phenotypic values per trait were visualized using ggplot2 (Wickham 2016) and verified using the Shapiro–Wilk normality test with the Agricolae R package (de Mendiburu and Yaseen 2020). Heritability and BLUEs were calculated using the R packages “statgenSTA” and “dplyr” (Wickham et al. 2023).

Prior to statistical analysis, genotypes represented by fewer than five plants were removed from the dataset. Outliers were identified based on standardized residuals using the function outlierSTA() implemented in *statgenSTA* and removed before refitting the model.

Best linear unbiased estimates (BLUEs) for each genotype were obtained using a linear model fitted with the fitTD() function assuming a resolvable incomplete block design (“res.ibd”). The model used for the analysis was:

$${y}_{ijk}={\mu +R}_{i}+(R:S{)}_{ij}+{G}_{k}+{\varepsilon}_{ijk,}$$

where yᵢⱼₖ is the observed phenotypic value for genotype k in the j-^th^ tank within the i-^th^ replicate, μ is the overall mean, Rᵢ is the fixed effect of the i-^th^ replicate (RepID), (R:S)ᵢⱼ is the effect of the j-^th^ sub-block (Tank) nested within replicate, Gₖ is the fixed effect of the k-^th^ genotype (Accession_ID), and εᵢⱼₖ is the residual error. Residual errors were assumed to be independently and normally distributed with mean zero and variance σ^2^ₑ. Variance components were estimated using restricted maximum likelihood (REML). Broad-sense heritability (H^2^) on an entry-mean basis was calculated as:

$${H}^{2}=\frac{{\sigma}_{g}^{2}}{{\sigma}_{g}^{2}+\frac{{\sigma}_{e}^{2}}{{n}_{r}}},$$

where ${\sigma}_{g}^{2}$= genetic variance, ${\sigma}_{e}^{2}$= residual variance, and ${n}_{r}=2$ is the number of replicates per genotype. Variance components, BLUEs, and heritability estimates were extracted from the fitted model using the functions STAtoTD() and extractSTA() implemented in *statgenSTA*.

Kruskal–Wallis test (McKight and Najab 2010) was used for testing trait mean differences among accessions grouped by release status or spike type.

The correlation analysis was performed using the Hmisc R package (Harrell Miscellaneous), calculating Spearman coefficients because not all phenotypic data follow a normal distribution and lateral root phenotypes are ordinal variables.

### SNP markers, statistical analysis, test for geographic origin

SNP data used in the present study were obtained from exome sequencing reads generated in the framework of the WHEALBI project (Bustos-Korts et al. 2019; Bretani et al. 2020) (NCBI BioProject PRJEB53544). Before carrying out SNP calling, the quality of exome sequencing reads was assessed with FastQC https://www.bioinformatics.babraham.ac.uk/projects/fastqc/ (Andrews 2010) and adapter sequences were removed using Trimmomatic v0.36 (Bolger et al. 2014). Read ends showing a base quality below 20 were automatically trimmed using Trimmomatic v0.36 (Bolger et al. 2014). High-quality reads obtained after filtering were mapped against the Morex v2 reference (Monat et al. 2019) using bwa-mem 0.7.17 (Li 2013). Duplicated reads in the resulting BAM files of alignments were detected and marked using the MarkDuplicates command of Picard (http://broadinstitute.github.io/picard). SNP calling and short indel detection were carried out using GATK v.4.3.0.0 (https://www.broadinstitute.org/gatk/; McKenna) following GATK best practices. Briefly, BAM files were used to perform per-sample variant calls within the exome capture target space using the HaplotypeCaller tool of GATK and the resulting per-sample GVCF files were subsequently consolidated in a unique GATK database using the GenomicsDBImport tool. Finally, the joint genotyping of the whole panel of samples was carried out using the GenotypeGVCFs tool to generate raw variant calls, which were further filtered by removing sites with > 50% missing genotype calls, QUAL < 30 and MAF < 0.05. After applying the aforementioned hard-filters, the resulting set of variants, which contained more than 537,097 SNPs, was used for subsequent genome-wide association analyses.

All heterozygous markers were converted to missing data by filtering markers that presented a percentage of missing data greater than Q3 + 1.5(Q3-Q1) (Q3 = third quartile, Q1 = first quartile). All missing data were imputed using Beagle (B L Browning and Zhou 2018). The population structure was computed on the 400 genotypes for which we had complete phenotypic and genotypic data using Admixture (Alexander et al. 2009) that allowed us to identify *K* = 9 subpopulations. Finally, markers were filtered for MAF > 0.05% and pruned for *R*^2^ = 0.99 using Plink (Purcell et al. 2007) which allowed us to select 537,097 markers used for GWAs. GWA was performed using the R package GAPIT (Wang and Zhang 2021) with the multi-locus mixed model method (MLMM) (Segura et al. 2012). Kinship, calculated using Tassel5 (Bradbury et al. 2007) and population structure were used as covariates. The proportion of phenotypic variance explained (PVE) by single QTL and globally for a trait were computed as a linear model; only the most significant marker for each QTL was considered when computing the global trait PVE. The genetic effect of each QTL was estimated by calculating the percentage difference between the average phenotypic values of the two alleles, relative to the overall mean.

To test for phenotypic differences between different geographic origins, a model Y = Origin + RowType + ReleaseStatus was tested using the lm() function in R. This was followed by a Tukey’s honest significant dfference (HSD) post hoc test applied to the corresponding aov object to assess pairwise differences among factor levels.

### Signature of selection detection

Genomic regions showing signatures of selection during barley domestication and breeding were identified by calculating population differentiation (Fst) and diversity reduction index (π wild/π domesticated, DRI), in pair between the three main germplasm groups (wild accessions, landraces, and cultivars as classified in the collection passport). Fst measures genetic differentiation between populations, capturing variation within and between groups while DRI quantifies reductions in genetic diversity resulting from processes such as selection and population bottlenecks. Fst was calculated for each pair of germplasm groups as previously described (Weir and Cockerham, 1984) with vcftools 0.1.16 (Danecek et al. 2011) using windows of 400 SNPs and a sliding step of 1 SNP. To calculate DRI, nucleotide diversity (*π* = Pi) at the polymorphic sites was estimated for each gene as the average of 50 consecutive genes (not limited to genes with SNPs) centered around the gene of interest using vcftools 0.1.16. To identify the chromosomal regions showing signatures of selection, minimizing the number of false positives, two thresholds were chosen: for DRI and Fst, each metric signal above the pair-based 95th or the 99th percentile was scored as a soft or hard sweep, respectively.

### QTL supporting interval, QTL alignment, and identification of candidate genes

Linkage disequilibrium decay at each QTL was investigated using Haploview (Barrett et al. 2005) in order to define QTL supporting intervals. A QTL supporting interval was defined as the marker interval around the top marker–trait association signal where markers resulted in LD (*r*^2^ > 0.3). To facilitate the comparative analysis of QTLs identified in the present study (Table S3) with those from other studies, a comprehensive list of QTLs for root traits in barley was compiled (Table S4) through a literature search employing the keywords “QTL and/or GWAS + barley + root.” QTL positions were aligned using the barley Morex v2 genome reference (Monat et al. 2019). The positions were determined based on the best available information from individual studies, including the physical positions of QTL flanking markers. The aligned QTL positions are presented in Table S3. Of the 106 detected QTLs, those QTLs with PVE and genetic effect greater than 5% were considered as major QTLs and underwent candidate gene analysis. At each major QTL, a candidate gene search was conducted within a genomic region defined by markers in linkage disequilibrium with *r*^2^ ≥ 0.3 with the locally most trait-associated marker. Genes were considered as candidates based on their molecular, biological and functional relevance to the traits under investigation as obtained from annotation of the closest orthologs in *Arabidopsis*, rice, maize and wheat, and from transcriptional profile. Root expression data were retrieved from BarleyExpDB: The Barley Expression Database, projects PRJEB14349 and PRJEB13621. The R package geneHap R (Zhang et al. 2023) was used for computing haplotypes in the coding sequence of selected candidate genes. Eventually, only genes included in the QTL supporting interval, expressed in the relevant root tissue or organ and with haplotype variants with at least one allele characterized by sequence variation impacting amino acid sequence were discussed as candidate genes.

## Results

### Phenotypic variation for root traits in barley collection is large and linked with domestication and release status

**Fig. 1:**
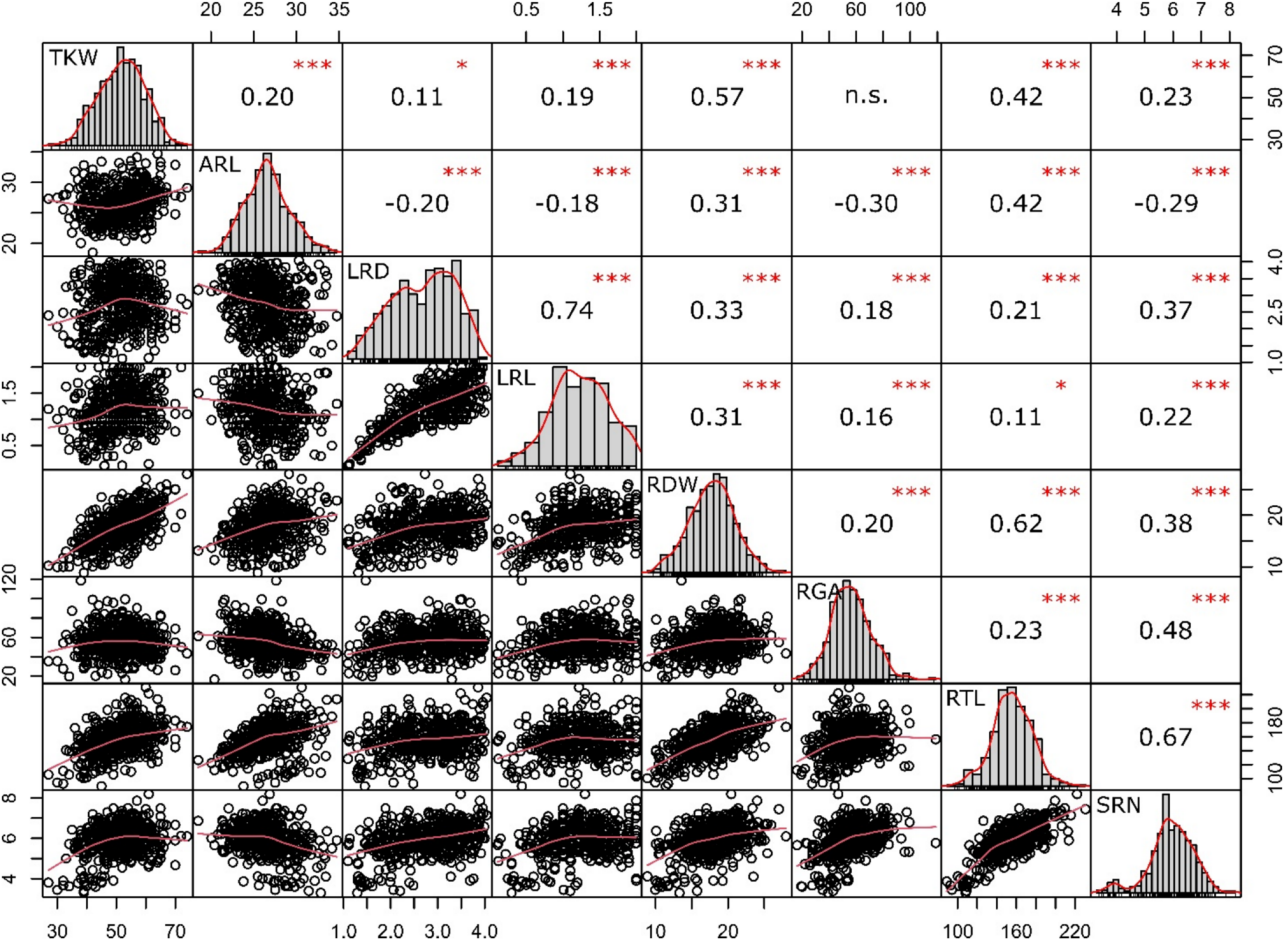
Distribution of root trait values and correlation among traits. Histograms of distribution frequency with smoothed regression lines (red) are reported on the diagonal; distribution clouds are reported on the left of the diagonal; correlation coefficients (Spearman) with the corresponding significance levels are shown on the right of the diagonal (*** = *P* < 0.001; * = *P* < 0.05; *ns* not significant)

**Fig. 2:**
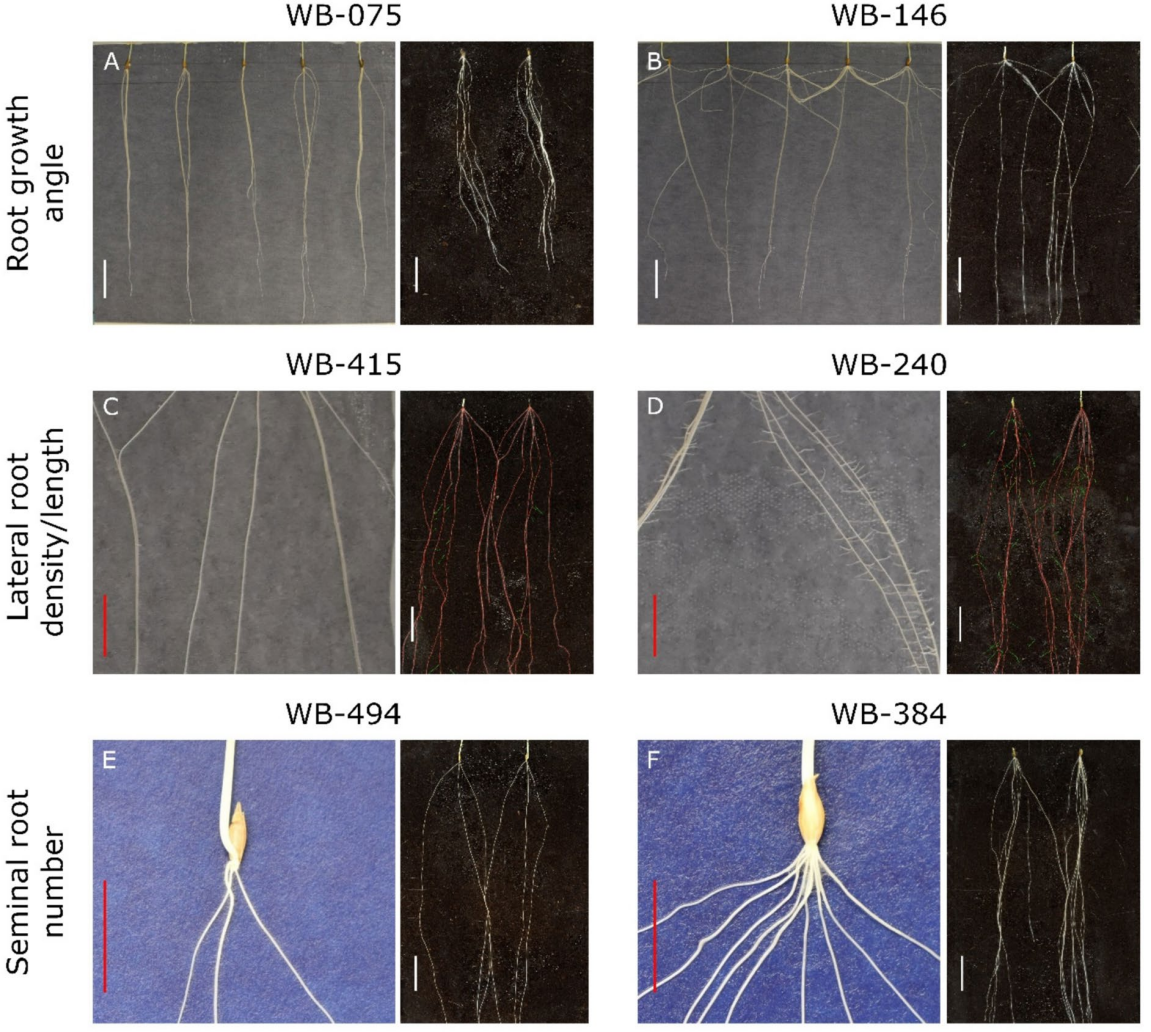
Examples of extreme variation in root system architecture traits observed in this study. For each accession, images from semi-hydroponic (left) and soil-filled rhizotron (right) are provided. **A, B** Variation in root growth angle (RGA). Accession WB-075 (landrace, RGA = 22.8°) exhibited a narrow root angle, whereas WB-146 (cultivar, RGA = 118.8°) showed a wide angle. **C, D** Variation in lateral root density (LRD) and lateral root length (LRL). WB-415 (landrace, LRD = 1.11; LRL = 0.11 score value) and WB-240 (cultivar, LRD = 3.96; LRL = 2.00 score value) showed minimal and maximum values, respectively, for LRD and LRL. **E, F** Variation in the number of seminal roots. WB-494 (wild, 3.29 seminal roots) and WB-384 (landrace, 8.20 seminal roots) showed the minimum and maximum phenotypes, respectively. Values reported in this legend correspond to mean phenotypic values for the respective accessions (all phenotypic data are reported in Supp. Tab S2). Scale bars: red = 2.5 cm; white = 5.0 cm

Phenotypic data for seven root traits (Table 1 and Table S1, S2) were collected from 448 barley accessions at the seedling stage (2 weeks after germination) grown in semi-hydroponics. All root traits showed an approximately normal distribution (Shapiro–Wilk normality test, *P* = ns. Figure 1). A wide range of phenotypic variation was observed across traits (coefficient of variation ranging from 10.2% for ARL to 34.3% for LRL. Table 1). Heritability ranged from *h*^2^ = 0.70 for Root dry weight (RDW) to 0.88 for SRN (Table 1). The observation of high *h*^2^ (0.88) for SRN is in line with the hypothesis that the number of seminal root primordia is largely determined already in the dormant seed (Luxová 1986); this makes SRN less affected by post-germination experimental factors or perturbation as compared to other root traits. Examples of accessions showing extreme phenotypes for SRN, root growth angle (RGA), lateral root density (LRD), and LRL are shown in Fig. 2. To verify whether phenotypes observed in semi-hydroponics were maintained under soil conditions, a subset of accessions with the extreme phenotypes (four lines for both RGA and SRN and six lines for lateral root density and length) was evaluated in soil-filled rhizotrons. For LRD, RGA and SRN, the selected lines maintained the ranking previously observed, with the lines selected for having the highest values in semi-hydroponics showing significantly higher values (*P* < 0.05) than those selected to have the lowest values (Fig. 2, Fig. S2, Table S5). For LRL, however, the ranking of the lines was not conserved between semi-hydroponics and rhizotron systems (Fig. S2 and Table S5).

Strong positive correlations (*r* > 0.5) were observed between LRL and LRD, SRN and root total length (RTL), and RTL and RDW (Fig. 1). A relatively weak negative correlation was detected between ARL and both RGA and SRN (*r* = − 0.30 and − 0.29, respectively; Fig.). The correlation between root traits and thousand kernel weight (TKW) was also tested. Only RDW and RTL showed a relatively high positive correlation with TKW (*r* = 0.57 and 0.42, respectively), whereas the correlation with other root traits was negligible (ranging from *r* = 0.11 with LRD to *r* = 0.23 with SRN, all *P* < 0.05), or not significant for RGA (Fig. 1).

**Fig. 3:**
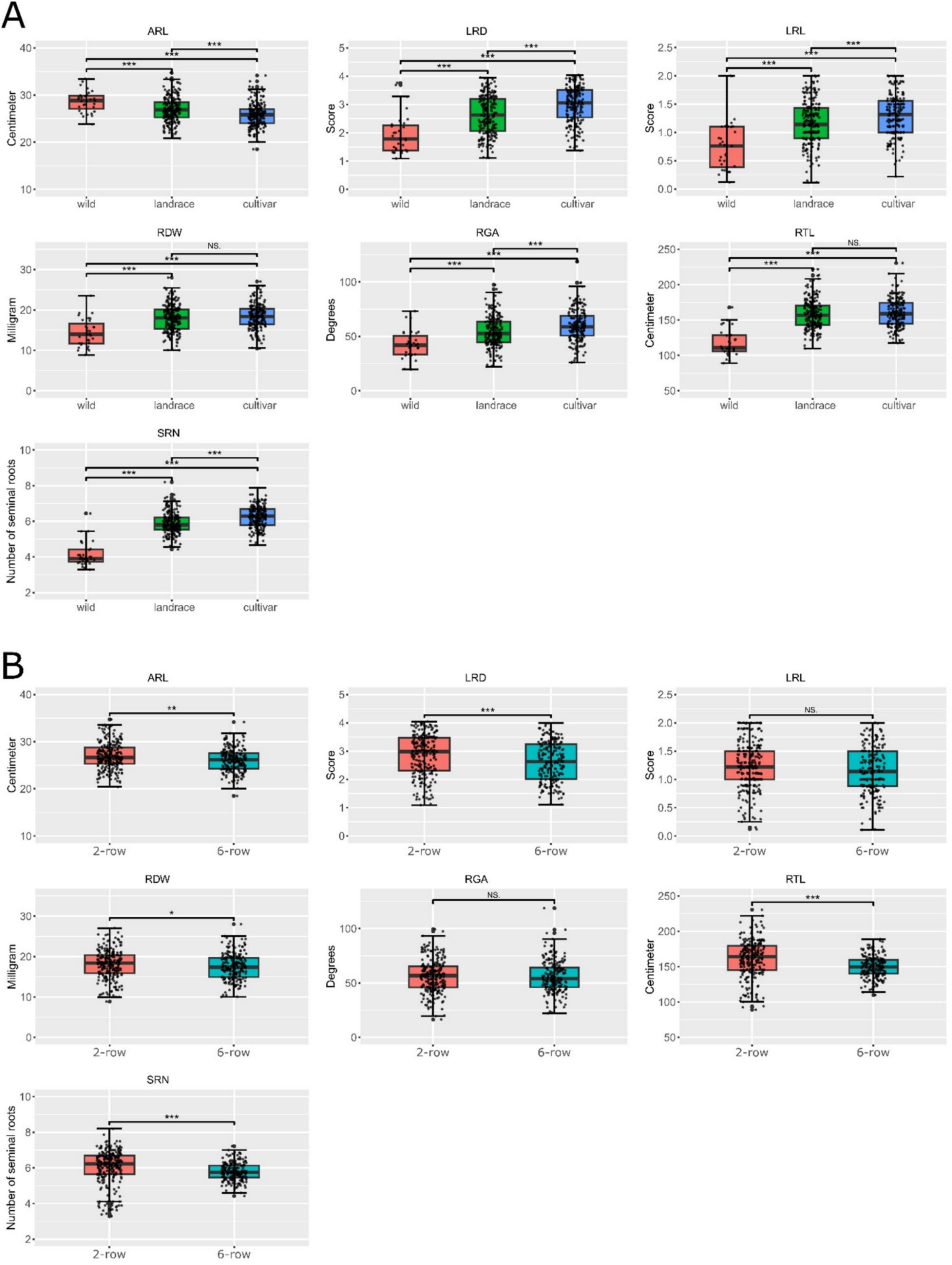
Root traits values analyzed by row type and release status. **A** Analysis by accession release status, namely wild accession, landrace or cultivar, and **B** Row type, namely 2- or 6-row. Horizontal axes represent release status or row-type; vertical axes represent phenotypic values. *ARL*, average root length; *LRD*, lateral root density; *LRL*, lateral root length; *RDW*, root dry weight; *RGA*, root growth angle; *RTL*, total root length; *SRN*, number of seminal roots. Significance based on Kruskal–Wallis test: *** = 0.001; ** = 0.01; * = 0.05

Significant differences in mean root trait values were observed when barley accessions were grouped by release status, namely wild accession, landrace and cultivar (Fig. 3A and Table S6). Specifically, SRN increased from 4.2 roots in wild accessions to 5.9 in landraces and 6.2 in cultivars (all contrasts, *P* < 0.01). A similar increasing trend was observed for RGA (42.9, 54.3 and 60.3 degrees. All contrasts, *P* < 0.01) and LRD (1.94, 2.62 and 3.0. All contrasts,* P* < 0.01). On the contrary, ARL decreased from wild to landrace to cultivar, from 28.7 to 26.9 to 25.8 cm, respectively (all contrasts, *P* < 0.01). For RDW and RTL, significant differences were detected only between wild and domesticated (landraces and cultivars) accessions, with domesticated accessions showing significantly higher values (Table S6). Changes of RSA throughout domestication and breeding, as detected in this study, are summarized in Fig. 6 and discussed further in Discussion. When classified by row type, two-row accessions showed higher mean values for ARL, RDW, RTL and SRN than six-row accessions, while no differences were observed for the other traits (Fig. 3B and Table S6).

### Genome-wide association study for RSA traits identified 106 QTLs

**Fig. 4:**
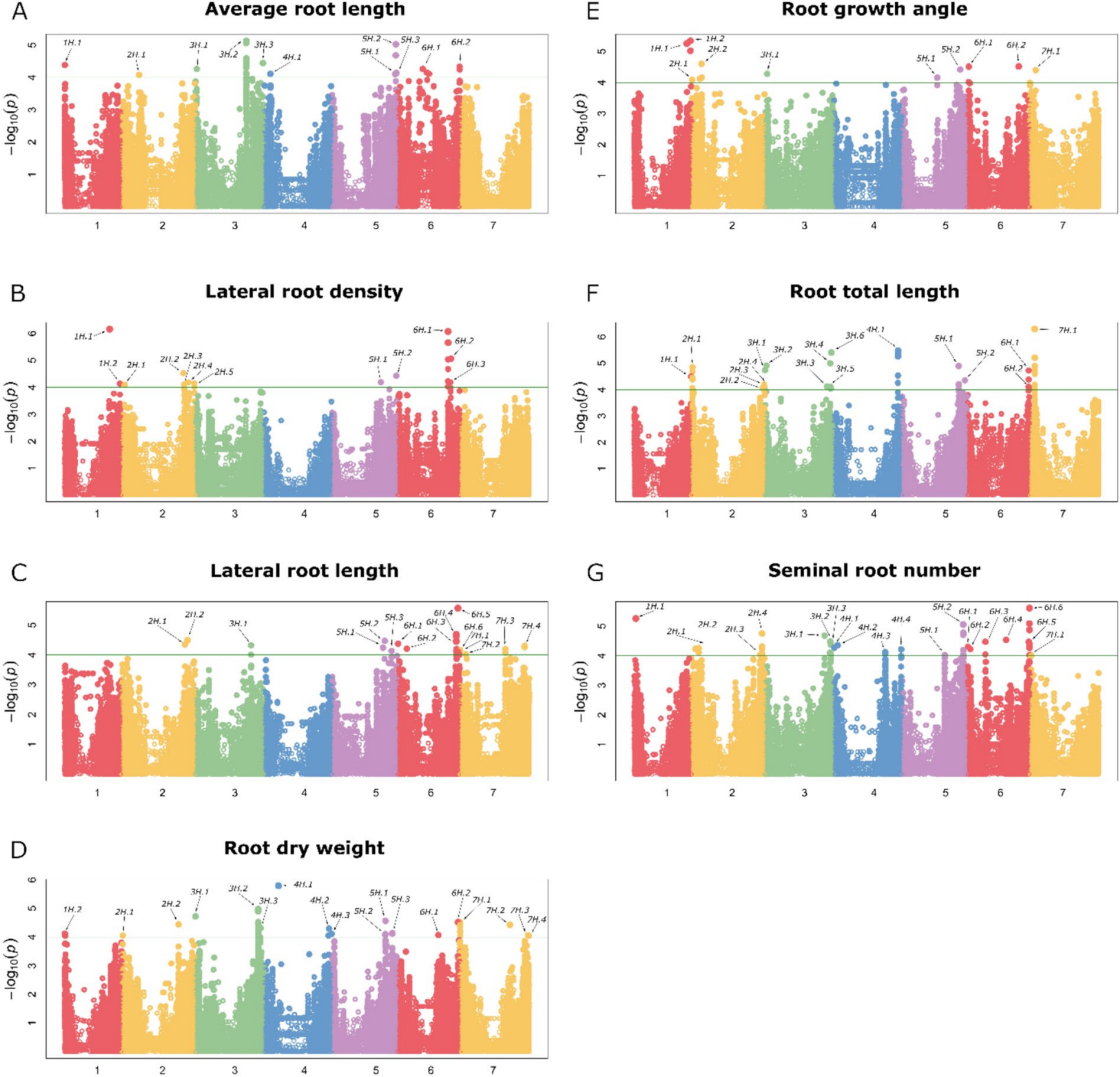
Genome-wide association mapping results for seminal root traits. For each plot (from **A** to **G**), horizontal axes represent the seven barley chromosomes; vertical axes represent the − log_10_(P-values) of marker–trait association. The horizontal green line shows the probability threshold for association (value − log_10_(P) = 4.00). All QTLs are labeled. For each QTL, further information is available in Table S3

A total of 537,097 SNP markers were used for GWAS, identifying 344 marker–trait associations (MTAs) (Fig. 4 and Table S3), which corresponded to 106 QTLs. Detailed information for all QTLs is summarized in Table S3. Only major QTLs (34 out of 106 QTLs) with proportion of variance explained (PVE) > 5% and genetic effect > 5% of the trait mean value (Table 2) will be discussed in detail. All 11 QTLs identified for ARL explained 41% of the PVE. The strongest were *qARL-1H.1, qARL-3H.1* and *qARL-5H.1,* with PVE values of 11.7%, 14.7% and 12% PVE and genetic effects of 8.0%, 6.3% and 8.1% of ARL mean value, respectively. Twelve QTLs for LRD explained 26% of the PVE. The strongest LRD QTLs were *qLRD-1H.2, qLRD-2H.3, and qLRD-5H.1* with PVE values of 5.5%, 5.6%, and 11.2% PVE, and genetic effects of 24.5%, 20.2%, and 26.2% of LRD mean value, respectively. For LRL, 16 QTLs explained 32% of the PVE. Three LRL QTLs explained more than 5% PVE: *qLRL-5H.1*, *qLRL-5H.2,* and *qLRL-7H.4*, with genetic effects of 19.6%, 22.6%, and 19.6% of LRL mean value, respectively. *qLRL-6H.5,* the QTL with the greatest genetic effect, accounted for 24.6% of the trait mean value and explained 3.5% of the phenotypic variation. Nineteen QTLs for RDW explained 54% of the PVE, with 8 QTLs showing PVE > 5%. The highest genetic effects were estimated for *qRDW-2H.2, qRDW-6H.1* and *qRDW-6H.2* affecting root biomass mean value by 15.2%, 16.9% and 15.9%, respectively. For RGA, 10 QTLs globally explained 35% of the PVE. The strongest RGA QTLs, *qRGA-2H.2* and *qRGA-6H.2,* explained 10.2% and 6.5% PVE, with genetic effects of 30.9% and 7.2% of the trait mean value, respectively. Seventeen QTLs for RTL explained 47% of the PVE. The strongest RTL QTLs, *qRTL-2H.2, qRTL-4H.1* and *qRTL-5H.1* explained 9.0%, 11.5% and 7.1% of PVE, with genetic effects corresponding to 15.9%, 14.1% and 16.0% of RTL mean value, respectively. SRN showed the greatest number of QTLs (21) and the highest global PVE (61%). The strongest SRN QTLs, *qSRN-4H.1*, *qSRN-4H.2* and *qSRN-6H.3* explained 25.6%, 17% and 23% of the PVE and had genetic effects of 14.5%, 14.7% and 13.7% of trait mean values (approx. 0.8–0.9 seminal roots), respectively.

**Table 2:** Major QTLs for seminal root traits identified in this work and gene model (Morex V2) of selected candidate genes with their molecular function

*QTL*	Most associate marker	P value	R^2^ (%)	Effect (%)	Candidate genes*	Molecular function
*qARL-1H.1*	S1H_2205412	4.12E-05	12.00	8.00	*HORVU.MOREX.r2.1HG0000900; HORVU.MOREX.r2.1HG0000910*	Pectin lyase-like superfamily protein; Jasmonate induced protein
*qARL-3H.1*	S3H_13758247	5.40E-05	15.00	6.30		
*qARL-5H.1*	S5H_578310092	7.80E-05	12.00	8.10	*HORVU.MOREX.r2.5HG0439520*	F-box family protein
*qLRD-1H.2*	S1H_505521454	7.27E-05	5.00	24.50		
*qLRD-2H.3*	S2H_573139845	7.85E-05	6.00	20.20	*HORVU.MOREX.r2.2HG0151020*	cotton fiber protein
*qLRD-5H.1*	S5H_447309122	6.41E-05	11.00	26.20		
*qLRL-5H.1*	S5H_467082368	5.81E-05	10.00	19.60		
*qLRL-5H.2*	S5H_482511141	3.41E-05	10.00	22.60	*HORVU.MOREX.r2.5HG0405780*	WPP domain-interacting protein 1
*qLRL-7H.4*	S7H_593343837	5.04E-05	7.00	19.20		
*qRDW-1H.2*	S1H_4636159	9.00E-05	6.00	6.60		
*qRDW-2H.2*	S2H_520884818	3.61E-05	14.00	15.20		
*qRDW-3H.1*	S3H_1285697	1.90E-05	7.00	9.50		
*qRDW-4H.2*	S4H_595144176	5.01E-05	17.00	12.90		
*qRDW-5H.1*	S5H_489011807	2.71E-05	11.00	8.70		
*qRDW-5H.3*	S5H_552156848	7.19E-05	6.00	7.50		
*qRDW-6H.1*	S6H_374719860	8.50E-05	5.00	16.90		
*qRDW-6H.2*	S6H_554225959	3.03E-05	11.00	15.90		
*qRGA-2H.2*	S2H_92247749	2.51859E-05	10.20	30.90	*HORVU.MOREX.r2.2HG0100950*	Glycosyltransferase
*qRGA-6H.2*	S6H_472869449	3.05029E-05	6.50	7.20	*HORVU.MOREX.r2.6HG0503680*	Tubby-like F-box protein
*qRTL-2H.1*	S2H_10181627	1.41506E-05	6.50	12.70		
*qRTL-2H.2*	S2H_655304719	8.67379E-05	9.00	15.90		
*qRTL-3H.3*	S3H_567210093	7.56354E-05	11.40	9.60		
*qRTL-4H.1*	S4H_591106805	3.30189E-06	11.50	14.10		
*qRTL-5H.1*	S5H_522759365	1.27578E-05	7.10	16.10		
*qRTL-5H.2*	S5H_579152000	4.45038E-05	18.60	12.50		
*qRTL-6H.1*	S6H_564047997	1.8853E-05	9.10	14.10		
*qRTL-7H.1*	S7H_46610184	5.09819E-07	6.80	13.90		
*qSRN-2H.1*	S2H_36837060	5.86E-05	5.70	7.90		
*qSRN-2H.3*	S2H_630408337	9.28E-05	6.60	6.20		
*qSRN-3H.1*	S3H_545852404	2.08E-05	7.80	10.70		
*qSRN-4H.1*	S4H_6264721	5.35E-05	25.60	14.50	*HORVU.MOREX.r2.4HG0278070*	Kinesin-like protein
*qSRN-4H.2*	S4H_36621909	4.40E-05	17.00	14.70	*HORVU.MOREX.r2.4HG0284880*	WD40-repeat protein
*qSRN-5H.2*	S5H_563800517	8.72E-06	9.60	6.20	*HORVU.MOREX.r2.5HG0433960*	Exocyst complex component EXO84B
*qSRN-6H.3*	S6H_167812278	3.38E-05	23.00	13.80	*HORVU.MOREX.r2.6HG0477060*	Subtilisin-like protease

Chromosomes are reported in the QTLs’ names

^*^See Materials and methods for selection of candidate genes. All candidates encompassing major QTLs regions are listed in Table S10

### QTL overlaps in this and other studies

Overlaps between QTLs for different RSA traits were observed in five instances (Table S3). On chromosome 1H, *qARL-1H.1* and *qRDW-1H.1* overlapped (at marker S1H_2205412), showing the same direction of genetic effect (i.e., the same marker allele was associated with higher ARL and RDW phenotypic values). On chromosome 3H, *qRDW-3H.1* and *qRTL-3H.1* overlapped at marker S3H_1285697, also showing the same effect direction. Additionally, *qRDW-3H.3* and *qRTL-3H.4* on chromosome 3H (markers S3H_598175765 and S3H_598175767) overlapped with the same effect direction. The same pattern was observed for *qLRL-6H.5* overlapping with *qRDW-6H.2,* and for *qLRL-7H.1* with *qRDW-7H.1*. In most cases, the direction of QTLs’ effects was consistent with the expected ontogenetic and biometrical connections between traits (e.g., higher ARL and/or RTL should result in higher RDW). One exception was the overlap involving *qRTL-4H.1* and *qRDW-4H.2,* where the minor SNP allele increased RTL while decreasing RDW. However, it is important to note that in this case the two QTLs only partially overlapped with their supporting intervals. QTLs for seminal root traits from other studies are listed in Table S4; their map coincidence with QTLs from this study is detailed in Table S3 and discussed in the Discussion section.

### QTLs for seminal root traits co-mapped with domestication and selection sweep chromosome regions

**Fig. 5:**
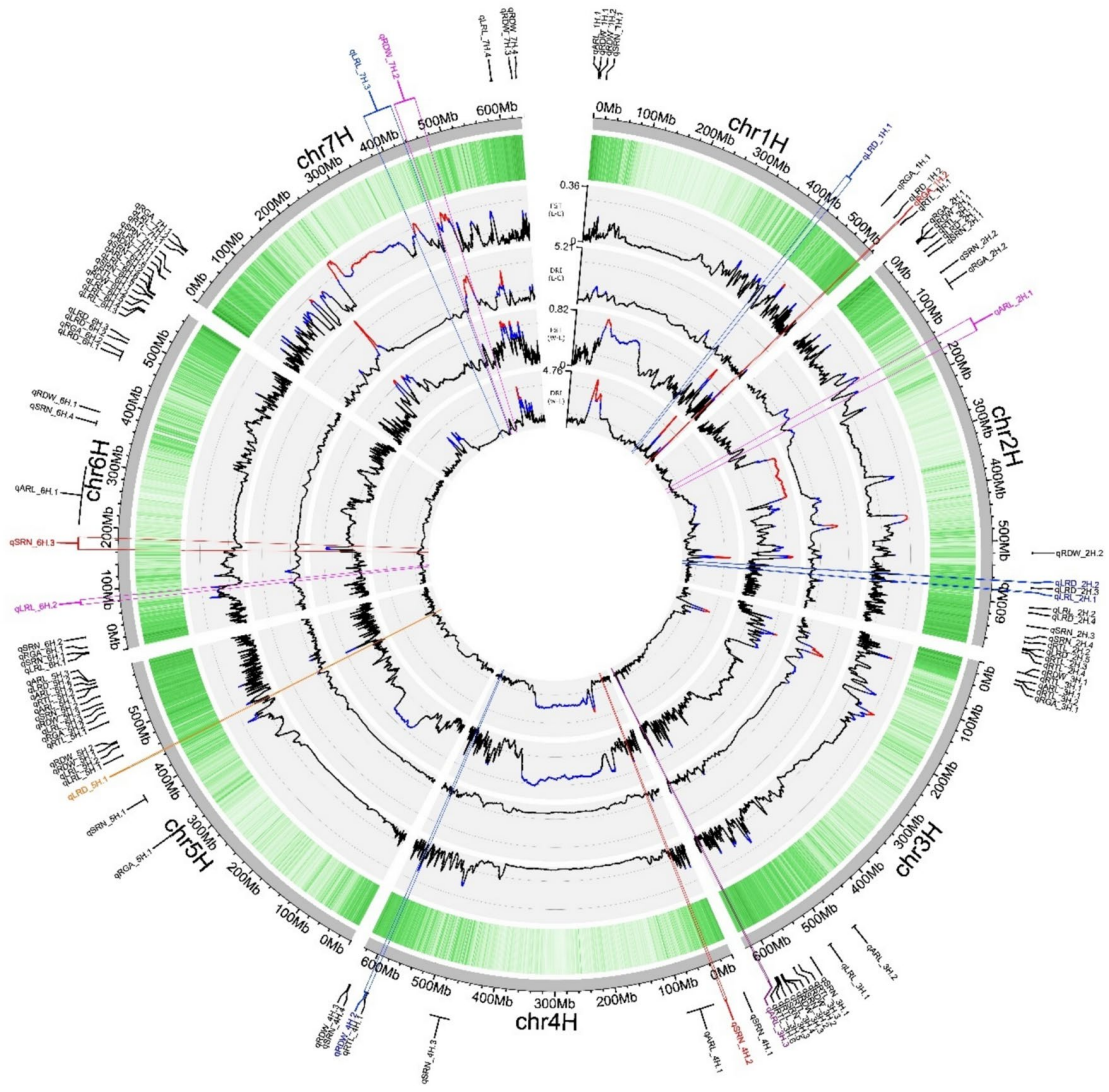
Circos plot of signature of selection obtained by Diversity reduction index (DRI) and Fst, and comparison with SNP and QTL distribution. From the center: DRI wild–landrace (w–l), Fst w–l, DRI landrace–cultivar (l–c), Fst l–c, SNP distribution (green heatmap, with darker regions indicating higher SNP density), chromosome physical map, QTL position. For DRI and Fst plots, regions highlighted in blue represent the pair-based 95th percentile, while regions highlighted in red indicate the pair-based 99th percentile. QTLs highlighted in red co-map with regions identified in the Fst w–l analysis, while those in purple correspond to regions from the DRI w–l analysis. QTLs in blue align with significant regions identified in the Fst l–c analysis, whereas those in orange are linked to the DRI l–c analysis. QTLs that align with regions identified in multiple analyses are shown in magenta

Analysis of population differentiation (Fst) and diversity reduction index (π wild/π domesticated, DRI) enabled us to identify chromosome regions showing differentiation and selection between wild, landrace, and cultivar accessions (Fig. 5. Table S7, S8 and S9). The strongest signals were observed between wild accessions and landraces across most chromosomes, except for chromosome 5 and 6 (Fig. 5). Chromosome 7 exhibited the greatest genetic divergence between wild–landrace and landrace–cultivar comparisons, particularly in centromeric and peri-centromeric regions (Fig. 5. Table S7, S8 and S9).

Regions identified as domestication or selection sweep overlapped with root QTLs identified in this study. For wild–landrace Fst-identified regions, overlaps were observed with *qRGA-1H.2, qSRN-4H.2, qSRN-6H.3,* and *qRDW-7H.2*, whereas for DRI-identified regions, only *qARL-3H.3* was identified within a high-confidence region. For landrace–cultivar Fst-identified regions, eight QTLs overlapped with Fst peaks (*qLRD-1H.1, qARL-2H.1, qLRD-2H.2, qLRL-2H.1, qRDW-4H.2, qLRL-6H.2, qLRL-7H.3, qRDW-7H.2*), and four QTLs (*qARL-2H.1, qLRD-5H.1, qLRL-6H.2, qRDW-7H.2*) overlapped with DRI-identified regions, three of which were also highlighted in the Fst analysis (Fig. 5, Table S7, S8 and S9).

### Candidate genes for seminal root trait QTLs

Candidate gene searches were restricted to the 34 major QTLs (out of 106) showing a PVE ≥ 5% and genetic effect ≥ 5% of trait mean value (Table 2). A total of 533 genes located within the supporting LD intervals of these 34 QTLs and expressed in root tissues based on barley database information (Li et al. 2023) were considered as candidate genes and are listed in Table S10. These candidate genes were further analyzed for coding sequence variants (missense or nonsense) identified within the barley WHEALBI collection and other criteria (see Materials and Methods). Eleven candidate genes for RGA, SRN, ARL, LRD and LRL were prioritized and further discussed (Discussion and Suppl. Text1). Details regarding sequence variation and haplotypic/allelic effects for the same 11 candidate genes are provided in Fig. S3, and tissue expression is provided in Table S11.

## Discussion

### Root trait variation discovered in this study can support breeding for resilience

In this study, variation and genetic control of seminal root traits in barley were investigated. The length, number, and growth angle of axial roots are the main components of root spatial architecture and thus determine soil exploration capacity (Maqbool et al. 2022), whereas lateral roots constitute the main interface between the plant and the soil and therefore influence nutrient absorption efficiency (Pélissier et al. 2021). Additionally, in small seed cereals such as barley, seminal roots remain active throughout the entire plant life cycle and represent approx. 40% of final root dry weight, with the remaining 60% represented by crown (nodal) roots that originate later in development (Anderson-Taylor and Marshall 1983). For these reasons, and because previous studies have reported correlations between variation observed in seminal roots at the seedling stage and in adult plants under field conditions (Maccaferri et al. 2016; Ober et al. 2021), the information obtained in this study has the potential to impact barley breeding.

This study showed that barley accessions with remarkably extreme seminal root phenotypes are present in both cultivated and wild germplasm. For example, landraces WB-075 and WB-363 were found to develop highly vertical (i.e., steep) seminal root systems (Fig. 2, and Table S2 and S5) similar to the hypergravitropic root mutants *EGT1* and *EGT2* originally identified in a chemically mutagenized population (Talamè et al. 2008; Kirschner et al. 2021; Fusi et al. 2022). We initially investigated whether *EGT1* and/or *EGT2* might play a role in the root phenotypes of WB-075 and WB-363. However, no functional variation (i.e., amino acid substitutions) was identified at *EGT2* across the entire WHEALBI collection (data not shown). In contrast, *EGT1* showed sequence variation across the collection and was identified as candidate for one of the RGA QTL (*qRGA-6H.2*. Table S10). Nevertheless, the *EGT1* variants found in WB-075 and WB-363 were not predicted to be deleterious by standard bioinformatics tools (i.e., SIFT. Vaser et al. 2016) and were not associated with strong RGA effects based on GWAS or haplotype analysis. Thus, the root hypergravitropism shown by WB-075 and WB-363 likely stems from a polygenic effect, with *EGT1* and possibly *EGT2* playing only minor roles. WB-075 and WB-363 originated from Ethiopia and Egypt (Pasam et al. 2014), respectively, and the development of their hypergravitropic root systems may have been driven by the selective advantage of a steeper and deeper root architecture, which could be beneficial in terminal drought conditions (Lynch 2018; Voss-Fels et al. 2018b; Elakhdar et al. 2022). Curiously, an opposite (i.e., ‘hyper-shallow’) root phenotype was observed in WB-146 (Fig. 2), WB-147 and WB-148, which originated from Far East Asia (Japan or Korea. Table S2). WB-146 is a formally bred cultivar selected in Japan and no information is available on the environmental water status where selection took place. However, WB-146 was already identified in an independent study as characterized by a pale leaf phenotype due to a mutation in *HUS/cpSRP43* (*Happy under the sun/ Chloroplast Signal Recognition Particle 43*) (Rotasperti et al. 2022). *HUS/cpSRP43* encodes for a stromal chaperon involved in the uploading of antenna proteins involved in light perception. Light perception is one of the mechanisms governing the gravitropic response in plants (Muthert et al. 2019), suggesting a potential mechanism underlying the shallow root growth of WP-146.

The two cultivars with the highest lateral root density and length, namely WB-167 and WB-240, originated from environments characterized by high humidity and rainfall (Bustos-Korts et al. 2019). Patterning of lateral roots is governed by water availability with water promoting the formation and growth direction of lateral roots (Orman-Ligeza et al. 2018; Giehl and von Wirén 2018; Placido et al. 2020; Kou et al. 2022; Scharwies et al. 2025). Thus, it is conceivable that genotypes selected in high soil humidity environments exhibit a relaxed control of lateral root growth. Indeed, a rapid lateral root developmental response to water and nutrient availability has been identified as a key trait in root ideotypes suited for sustainable irrigated cropping systems (Schmidt and Gaudin 2017).

Significantly higher ARL values were observed for domesticated accessions WB-108, WB-263 and WB-294, which originated from the Middle East region typically characterized by arid environments (Syria, Iraq, Turkey, Saudi Arabia) (Table S2). The same accessions showed significantly lower SRN values. These observations are in line with the hypothesis that plants investing in root length rather than the number of seminal roots have higher water uptake potential from deeper layers (McCulley et al. 2004; Lynch 2018; Zhang et al. 2024). Conversely, accessions with shorter roots and a greater number of seminal roots originated in high input regions, such as Northern and East Europe (Table S2), and thus are likely optimized for nutrient absorption from the upper soil layers.

In order to assess the stability of root phenotypes beyond semi-hydroponics, the lines exhibiting the most extreme phenotypes for LRD, LRL, RGA, and SRN in semi-hydroponics were tested in soil-filled rhizotrons. The ranking of the lines was almost perfectly consistent between the two conditions for LRD, RGA and SRN, supporting the robustness of semi-hydroponic system-based results for these traits and a prospective interest in real field conditions. Line rankings were not confirmed for LRL. The greater sensitivity of LRL to environmental conditions is not unexpected, as LRL is known to be strongly influenced by growing medium and nutrient availability (Lopez et al. 2022) which were indeed markedly different between the two growth environments.

### Root domestication syndrome extends beyond number of seminal roots and it is supported by the detection of selection sweeps at key loci

**Fig. 6:**
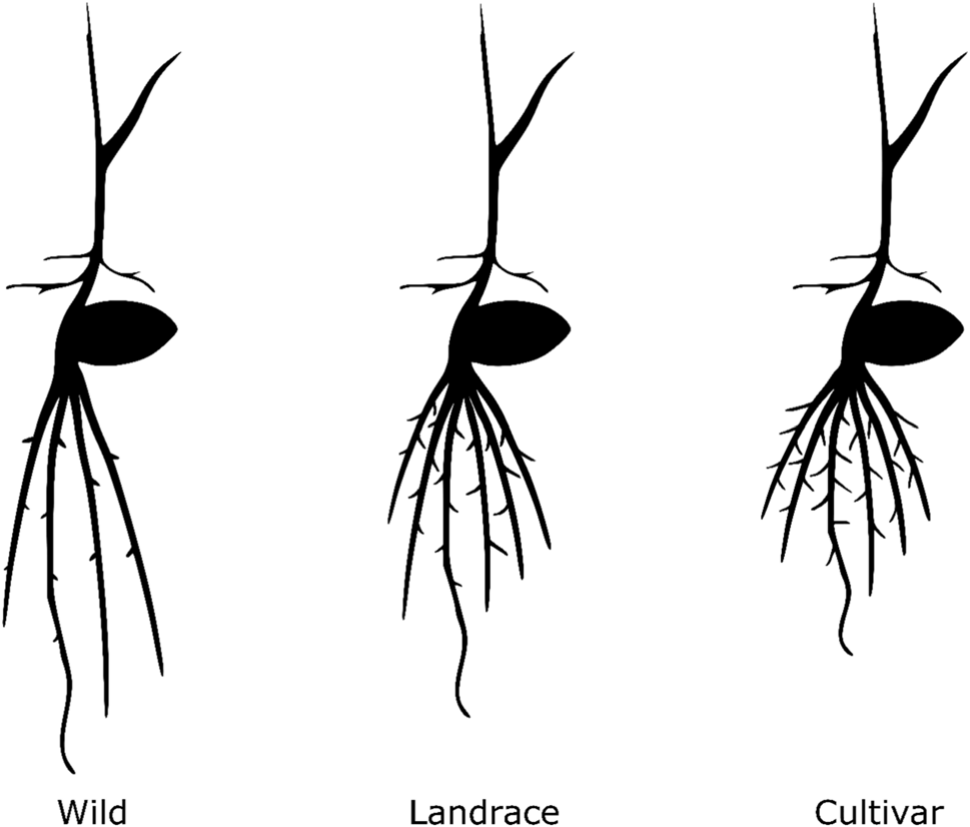
Effect of domestication and breeding on seminal root system architecture in barley. Each seedling scheme represents a release status (wild, landrace or cultivar) and was drawn based on the seminal root traits’ mean values for traits collected in this study (Table S2) or rounded to the closest unit (for number of seminal roots). Traits considered were average root length (ARL), number of lateral roots (LRN), length of lateral roots (LRL), root growth angle (RGA), number of seminal roots (SRN). Other seedling parts (e.g., seed, shoot, etc.) are not drawn to scale. The starting seedling image was obtained from BioRender (www.biorender.com)

The number of seminal roots was already recognized as affected by domestication and breeding in barley, wheat and maize, where it was shown to increase from wild accessions to landraces to cultivars (Grando and Ceccarelli 1995; Burton et al. 2013; Golan et al. 2018; Perkins and Lynch 2021). In maize, this increase was linked with improved mineral nutrients acquisition (Zhu et al. 2006; Perkins and Lynch 2021). In wheat, Golan et al. (2018) showed that two additional seminal roots were gained from wild to cultivated wheat; however, no difference was found in the number of seminal root primordia. Dormant primordia in wild wheat can reactivate upon dehydration and re-hydration of the young seedling, likely providing a survival mechanism under water stress events (Golan et al. 2018). Similarly to the above studies, our study strongly supports that the number of seminal roots is affected by domestication and breeding syndrome in barley. Furthermore, we extended such effects to other root traits (Fig. 6). We showed that barley gained approx. 1.7 seminal roots from wild accessions to landraces and further > 0.3 roots from landraces to cultivars. This change was mirrored in our experimental settings by a reduction in the average length of seminal roots from wilds to cultivars. Such evolutionary changes in number and length of seminal roots can be interpreted as a genetically controlled re-distribution of resources which took place as a consequence of the unconscious gradual selection of early farmers while adapting barley to different soil environments or management practices, namely heavier use of fertilizers, higher planting density, introduction or change of soil tillage, etc. At the moment, we do not have information on the difference in root primordia which could be compared with the information available in wheat (Golan et al. 2018). Additional changes were observed for root growth angle (gain of > 17° from wilds to cultivars) and lateral roots (83% increase in density and 45% increase in length, from wilds to cultivars), likely providing improved soil exploration efficiency in the upper soil layers in the domestic accessions, as suggested in maize (Ali et al. 2015) and in wheat (Roucou et al. 2018). Of course, the consistency of these phenotypes and QTL effects from controlled to field conditions needs to be verified for all traits, perhaps with the exception of SRN which is expected to be completely defined at germination.

The overlap between domestication sweep regions and root QTLs further supports our root domestication syndrome observations. Specifically, two major SRN QTLs (*qSRN-4H.2* and *qSRN-6H.3*) co-localized with domestication sweep chromosome regions (from wild accessions to landraces. Figure 5, Table S8), while a QTL for root growth angle (*qRGA-1H.2*) was found overlapping with a domestication region on chromosome 1 supported by Fst analyses (Fig. 5, Table S8). To the best of our knowledge, these regions were not identified before as domestication sweep loci.

Independently from root traits, different genes classified as candidates for domestication in former studies (Table S9; Pankin et al. 2018; Hill et al. 2021; Civáň et al. 2021) co-mapped with numerous domestication regions across all chromosomes in our analysis (Table S7 and S8). For example, at the beginning of chromosome 7H, we identified a selected region differentiating wild and landrace accessions in both DRI and Fst analyses, which harbored the gene *HORVU.MOREX.r2.7HG0604280,* formerly recognized as a domestication candidate (Pankin et al. 2018) (Table S7, S8 and S9) and which encodes a peroxidase highly expressed in root elongation and maturation zones (BarleyExpDB. http://barleyexp.com/).

*VRN1*, classified as domestication gene by Pankin et al. (2018) but not in our study, co-mapped with a QTL for root growth angle (*qRGA-5H.2.* Table S3, and S8). *VRN1* is already known to be involved in modulating flowering and root system in barley (Deng et al. 2015; Voss-Fels et al. 2018a).

### Barley root QTL positions are confirmed across studies

Many QTLs for seminal roots identified in this work co-mapped with QTLs identified in other studies (Robinson et al. 2016, Jia et al. 2019 and Abdel-Ghani et al. 2019). All overlaps are reported in Table S3. Interestingly, *RNQ3* and *RNQ5* identified by Robinson et al. 2016 co-map with *qSRN-4H.4* and *qSRN-6H.3,* two of the most important QTLs identified in our study. Furthermore, *qSRN-3H.3* co-maps with *QSRN2,* identified by Jia et al. (2019). Regarding RTL, the main QTL identified in our study, *qRTL-2H.2*, co-maps with *qTSRL4* identified by Jia et al. 2019, while other two QTLs, *qRTL-4H.1* and *qRTL-7H.1* co-map with *QTL-4H-7* and *QTL-7H-4*, respectively, identified by Abdel-Ghani et al (2019). For RGA, *qRGA-5H.2* co-maps with *qRSA15* (Jia et al. 2019); this QTL is near the *VRN-H1* gene that is involved in flowering and in modulation of root growth angle and root length in barley and wheat (Voss-Fels et al., 2018a). *qARL-4H.1* co-maps with *qASRL5,* formerly identified by Jia et al. (2019). Regarding RDW, seven QTLs identified by Abdel-Ghani (2019) co-map with QTLs for this trait. In particular, *qRDW-2H.2* and *qRDW-6H.1*, two of the most effective QTLs for this trait, are in the same region of *QTL-2H-6* and *QTL-6H-2*, respectively (Abdel-Ghani et al. 2019).

### Candidate genes at QTLs for root architecture traits

As far as RGA is concerned, two QTLs, namely *qRGA-2H.2* and *qRGA-6H.2,* were analyzed for candidate genes in detail. Both QTLs correspond to two previously identified meta-QTLs for root traits in wheat, *Root_MQTL_19* and *Root_MQTL_70, respectively.* Soriano and Alvaro (2019). Moreover, *qRGA-2H.2* co-maps with a syntenic region in rice in which *MQTL1-5* was identified for different root traits including root surface area (Daryani et al. 2022). At *qRGA-2H.2*, *HORVU.MOREX.r2.2HG0100950* revealed haplotype #6 with a significantly low RGA value (Fig. S3). This haplotype contains two unique missense SNPs at positions 2H:81,640,364 and chr 2H:81,641,333 causing F150V and D473H amino acid substitutions. *HORVU.MOREX.r2.2HG0100950* encodes a UDP-glycosyltransferase (UGT) that catalyzes the glycosylation of receptor molecules, including plant hormones such as auxin. Auxin gradient and concentration regulate various aspects of plant growth and development including root gravitropism. A decrease in auxin level in the root cap, caused by the overexpression of UGT genes catalyzing the glycosylation of IAA can lead to a loss of root gravitropic response (Woo et al. 2007; Tanaka et al. 2014). The confidence interval of *qRGA-6H.2* encompassed 14 high-confidence (HC) candidate genes including *EGT1 (HORVU.MOREX.r2.6HG0503680),* which was previously shown to be involved in controlling root gravitropism (Fusi et al. 2022), providing support to our QTL candidate gene analysis. *EGT1* haplotype #4, characterized by the amino acid substitution S306C, showed a significantly wider root growth angle than the other haplotypes (Fig. S3). Moreover, the known role of *EGT1* in influencing root angle suggests that natural genetic variation at this locus contributes to the observed phenotypic variation, further reinforcing its potential functional significance.

Regarding SRN, the strongest genetic effect QTL was *qSRN-4H.1,* which included *HORVU.MOREX.r2.4HG0278070*. This gene encodes an ARM-repeat superfamily protein and just two haplotypes are present in the WHEALBI collection (Fig. S3). An ARM-like protein known as ARABIDILLO was shown to affect root architecture by promoting root branching through the degradation of positive regulators of gibberellins in *Arabidopsis* (Mu et al. 2010; Nibau et al. 2011).

At *qSRN-4H.2*, which co-maps with the syntenic QTL *MQTL3-4* in rice (Daryani et al. 2022)*, HORVU.MOREX.r2.4HG0284880* exhibited three distinct haplotypes (Fig. S3). Haplotypes #2 and #3 shared five common missense mutations (4H:35,433,223, 4H:35,433,653, 4H:35,433,662, 4H:35,433,722, and 4H:35,433,794 corresponding to E281K, G424A, V427A, Q447R, and L471R amino acid substitutions) and had a significantly lower number of roots when compared with haplotype #1. This gene belongs to the WD40 protein family, which plays a key role in plant growth mechanisms mediating protein–protein interaction or protein-DNA interaction (Jain and Pandey 2018). WD40 proteins complex with members of MYB and bHLH transcription factor families and affect root growth in Arabidopsis by regulating the root epidermal cell patterning (Tan et al. 2021; Meng et al. 2024). In maize*, lateral rootless 1 (lrt1)* contains a WD40 domain and controls lateral root formation in primary and seminal roots (Baer et al. 2023). Furthermore, *lrt1* was shown to act upstream of *Rum1*, a maize gene involved in controlling both the number of seminal roots and lateral roots (Baer et al. 2023).

*qSRN-5H.2* co-maps with *Root_MQTL_62*, a meta-QTL associated with multiple root traits identified in wheat by Soriano Within the confidence interval of this QTL, we identified *HORVU.MOREX.r2.5HG0433960,* which showed three haplotypes affecting SRN. This gene encodes an exocyst complex component highly expressed in roots. Mutations in individual exocyst subunits result in root-hairless (short root hairs) phenotypes in maize and Arabidopsis (Hála et al. 2008; Fendrych et al. 2010). In rice *an EXO70-related gene co*ntrols the number of embryonic radicles, i.e., roots differentiated and emerging at the very initial phases of seed germination; the gene is also essential for extensive lateral root formation by modulating auxin homeostasis (Wang et al. 2024).

*qSRN-6H.3* co-maps with the syntenic region encompassing *MQTL2-3* identified in rice (Daryani et al. 2022); *qSRN-6H.3* includes *HORVU.MOREX.r2.6HG0477060*. At this gene, accessions carrying haplotypes #3 and #4 exhibited a significantly lower number of seminal roots. Haplotype #4 is characterized by four unique SNPs (6H:167,812,535, 6H:167,813,279, 6H:167,813,408, and 6H_167816514) resulting in V663A, V466M, T451I, and G87E amino acid substitutions. This gene encodes a serine protease that belongs to the subtilisin-like family and is known to be associated with the proteolytic activity in the extracellular matrix during the emergence of lateral roots (Neuteboom et al. 1999). Candidate genes for average root length, lateral root length and lateral root density QTLs are discussed in Supplementary text 1.

In this study, we showed that there is large, genetically controlled variation in seminal root traits relevant to breeding across barley accessions, including wild accessions, landraces and cultivars, and that this variation is at least partially maintained when moving from semi-hydroponic to soil-filled rhizotron systems. QTLs and candidate genes underlying root variation were identified providing a robust basis for advancing from QTL mapping to gene cloning. Additionally, these findings offer direct opportunities for crop improvement, as favorable alleles can be efficiently deployed through marker-assisted selection, although more information is still required in order to fully model the effect of different RSAs in different environments and to recruit, pyramid or edit the corresponding RSA genes. Ultimately, our results establish a translational framework linking root trait genetics to barley breeding by means of genomic and editing approaches.

## Supplementary Information

Below is the link to the electronic supplementary material.

Fig. S1. Seminal root system test images used as references for scoring lateral root density (LRD) and lateral root length (LRL). (A) Four-index scoring scale for LRD. (B) Three-index scoring scale for LRL. Scale bar = 2 cm. Detailed descriptions of the scoring indices are provided in Table S1.Supplementary file1 (JPG 3690 KB)

Fig. S2. Seminal root traits phenotypic values of selected lines grown in soil-filled rhizotrons. A) Number of lateral roots. B) Lateral root length (pixel). C) Root growth angle (°). D) Number of seminal roots. Different letters indicate statistically different values (P <0.05, Tuckey’s). Extreme accessions selected for each trait were as follows: RGA (accessions min: WB-075 and WB-363; max: WB-146 and WB-147), LRD/LRL (min: WB-075, WB-415 and WB-480; max: WB-240, WB-254 and WB-311) and SRN (min: WB-494 and WB-497; max: WB-240 and WB-384).Supplementary file2 (PDF 321 KB)

Fig. S3. Sequence variation and phenotypic effect for candidate genes. The significance levels of mean values in the violin plots correspond to 0.05, 0.01 and 0.001, indicated by *, ** and ***, respectively. For each haplotype, all SNPs in the coding sequence and the position based on reference Morex V2 are reported in the table below each violin plot. SNP positions that cause a protein change (missense or stop gain/lost) are marked with “*”.Supplementary file3 (PDF 2693 KB)

Supplementary file4 (DOCX 52 KB)

Supplementary file5 (XLSX 48246 KB)

Supplementary file5 (GZ 42512 KB)

## Data Availability

All data supporting this study are included in the article and/or in supporting materials. Genotypic data are uploaded as VCF file (WHEALBI_SNPs_400Accessions.vcf.gz).

## Abbreviations

ARL: Average root length
LRD: Lateral root density
LRL: Lateral root length
RDW: Root dry weight
RGA: Root growth angle
RTL: Total root length
SRN: Number of seminal roots
TKW: Thousand kernel weight
RSA: Root system architecture
GWAS: Genome-wide association studies
QTL: Quantitative trait loci
