# Supplementary figures and images for: Genetic dissection of root traits in barley identifies major QTLs and domestication signature
Source: Plant Cell Rep. 2026 May 23;45(6):174. doi: 10.1007/s00299-026-03852-3 (PMC13198497; doi:10.1007/s00299-026-03852-3)

A

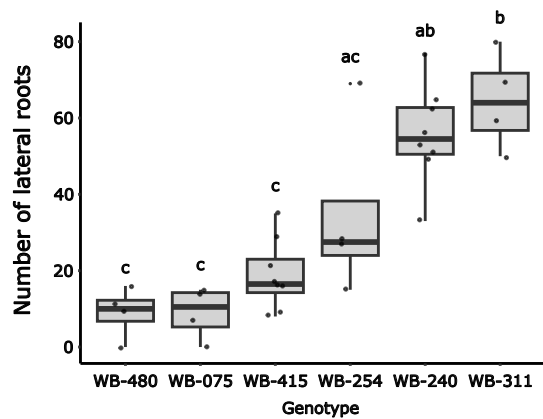

B

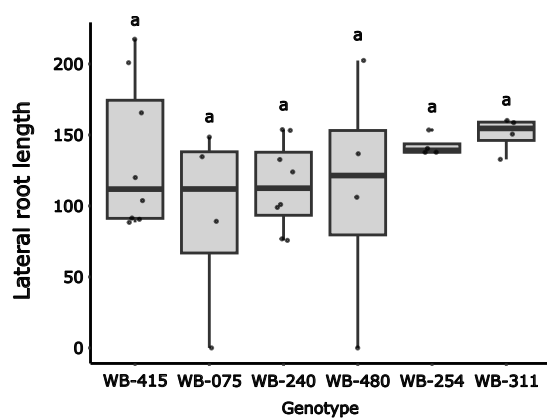

C

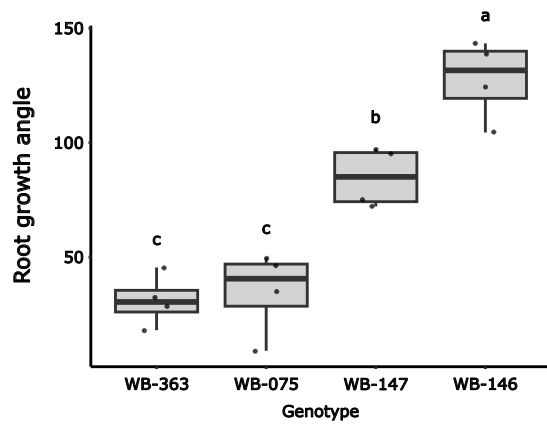

D

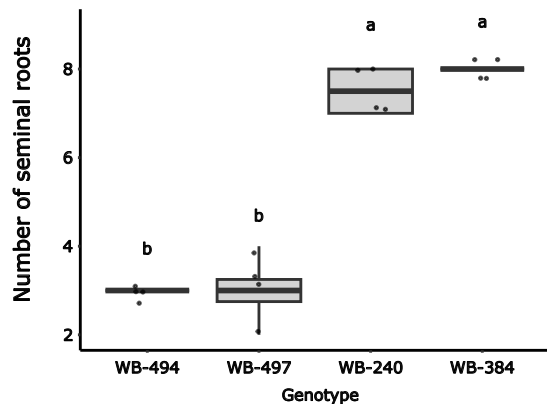

Supplement: Supplementary file 2 — Fig. S2. Seminal root traits phenotypic values of selected lines grown in soil-filled rhizotrons. A) Number of lateral roots. B) Lateral root length (pixel). C) Root growth angle (°). D) Number of seminal roots. Different letters indicate statistically different values (P <0.05, Tuckey’s). Extreme accessions selected for each trait were as follows: RGA (accessions min: WB-075 and WB-363; max: WB-146 and WB-147), LRD/LRL (min: WB-075, WB-415 and WB-480; max: WB-240, WB-254 and WB-311) and SRN (min: WB-494 and WB-497; max: WB-240 and WB-384).Supplementary file2 (PDF 321 KB) [file 299_2026_3852_MOESM2_ESM.pdf]
