# Supplementary material for: Genetic dissection of root traits in barley identifies major QTLs and domestication signature
Source: Plant Cell Rep. 2026 May 23;45(6):174. doi: 10.1007/s00299-026-03852-3 (PMC13198497; doi:10.1007/s00299-026-03852-3)
Supplement: Supplementary file 3 — Fig. S3. Sequence variation and phenotypic effect for candidate genes. The significance levels of mean values in the violin plots correspond to 0.05, 0.01 and 0.001, indicated by *, ** and ***, respectively. For each haplotype, all SNPs in the coding sequence and the position based on reference Morex V2 are reported in the table below each violin plot. SNP positions that cause a protein change (missense or stop gain/lost) are marked with “*”.Supplementary file3 (PDF 2693 KB) [file 299_2026_3852_MOESM3_ESM.pdf]

HORVU.MOREX.r2.1HG0000900 (qARL-1H.1)

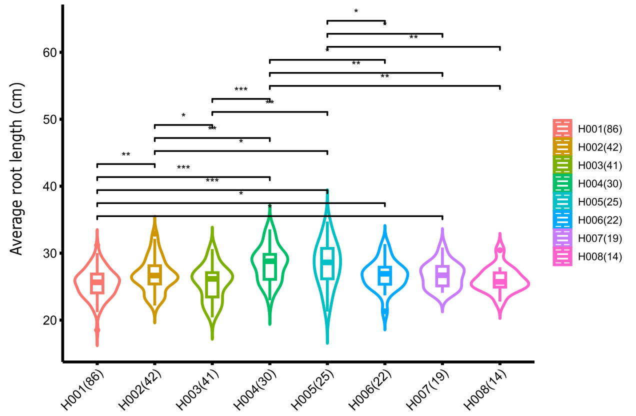

	2224685	2224702	2224719	2224762	2224774	2224795	2224880	2224881	2224902	2224912	2224930	2228515	2228564	2228568	2228576	2228592	2228604	2228660	2228668	2228669	freq
	*		*		*	*	*	*	*			*	*	*		*	*		*		
ALLELE	C/A	C/G	A/C	G/A	C/G	G/T	G/T	A/G	G/A	G/C	C/T	G/C	A/T	G/A	C/T	G/A	G/A	C/T	C/T	G/A	
H001	C	C	A	G	C	G	G	A	G	G	C	G	A	G	C	G	G	C	C	G	86
H002	C	C	A	G	C	G	G	A	G	C	C	G	A	G	C	G	G	C	C	G	42
H003	C	C	A	G	G	G	G	A	G	G	C	G	A	G	C	G	G	C	C	G	41
H004	A	C	A	G	C	G	G	A	G	C	C	G	A	G	C	G	A	C	T	G	30
H005	A	C	A	G	C	G	T	A	G	C	C	G	A	G	C	G	G	C	C	G	25
H006	C	G	A	A	C	T	G	A	A	C	C	G	A	G	C	G	G	C	C	G	22
H007	C	G	A	G	C	G	G	A	G	G	C	G	A	G	C	G	G	C	C	G	19
H008	C	C	A	G	C	G	G	G	A	C	C	C	T	A	T	A	G	C	C	G	14

HORVU.MOREX.r2.1HG0000910 (qARL-1H.1)

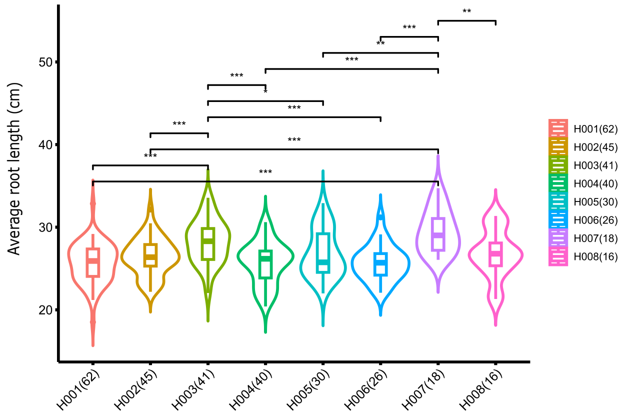

	2238315	2238498	2238510	2239218	2239315	2239358	2239400	2239522	2239529	2239532	2239533	2239539	2239959	2239972	2239973	freq
					*	*	*	*	*	*	*		*	*	*	
ALLELE	G/T	T/A	G/A	G/A	G/A	C/T	C/T	G/A	C/G	A/G	T/C	G/A	C/A	G/A	C/T	
H001	G	T	G	G	G	C	C	G	C	A	T	G	C	G	C	62
H002	G	T	G	G	G	C	C	G	C	A	C	G	C	G	C	45
H003	G	A	G	G	G	C	C	A	G	G	C	G	A	G	C	41
H004	G	T	G	G	G	C	T	G	C	A	C	G	C	G	C	40
H005	G	T	G	G	G	C	C	A	C	A	C	G	C	G	C	30
H006	G	T	G	G	G	C	C	G	C	A	C	G	C	A	C	26
H007	G	A	G	A	A	C	C	A	G	G	C	G	C	G	C	18
H008	G	T	G	G	G	C	C	A	C	A	C	A	C	G	C	16

HORVU.MOREX.r2.5HG0439520 (qARL-5H.2)

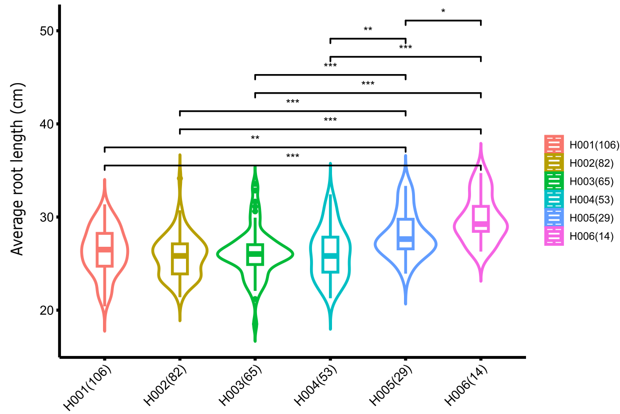

	578315494	578315578	578315592	578315605	578315613	578315665	578315676	578315725	578315743	578315765	578315767	578315833	578315845	578315848	578315860	578315932	578316057	578316096	578316116	578316137	578316160	578316244	578316273	578316286	578316316	578316332	578316435	578316473	578316480	578316547	578316585	freq
			*		*		*	*		*							*		*	*			*			*	*	*	*		*	
ALLELE	A/G	A/C	C/A	G/A	C/T	G/A	C/G	A/C	C/T	T/C	C/T	G/A	G/A	G/A	G/A	A/G	G/C	G/A	G/A	C/T	C/G	G/C	C/T	C/G	A/G	C/T	T/C	G/C	C/T	G/A	G/A	
H001	G	C	C	G	C	G	C	A	T	T	T	G	G	G	G	A	C	A	A	T	G	G	C	C	A	C	T	G	T	G	G	106
H002	A	A	C	G	C	G	C	A	C	T	C	G	G	G	G	A	G	G	G	C	C	G	C	C	A	C	T	G	C	G	G	82
H003	A	A	C	G	C	G	C	A	C	T	C	G	G	G	G	A	G	G	G	C	C	G	C	C	A	C	T	G	C	G	A	65
H004	A	A	C	G	C	A	C	A	C	T	C	G	G	G	G	A	G	G	G	C	C	G	C	C	A	T	T	G	C	G	A	53
H005	A	A	A	G	C	G	G	C	C	T	C	G	A	A	G	A	G	G	G	C	C	G	C	C	A	C	T	G	C	G	A	29
H006	A	A	C	A	C	G	C	A	C	T	C	G	G	G	A	A	C	A	A	T	G	G	C	C	A	C	T	G	C	G	A	14

HORVU.MOREX.r2.2HG0151020 (qLRD-2H.3)

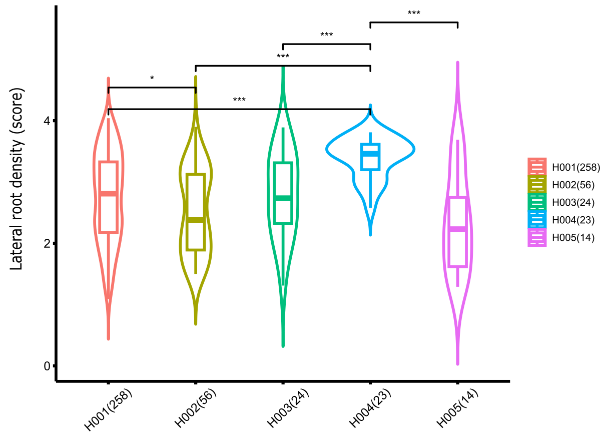

	572931098	572931234	572931283	572931415	freq
		*	*	*	
ALLELE	T/C	G/A	G/A	T/G	
H001	C	G	G	T	258
H002	T	G	G	T	56
H003	C	G	A	T	24
H004	C	A	G	T	23
H005	C	G	G	G	14

HORVU.MOREX.r2.5HG0405780 (qLRL-5H.2)

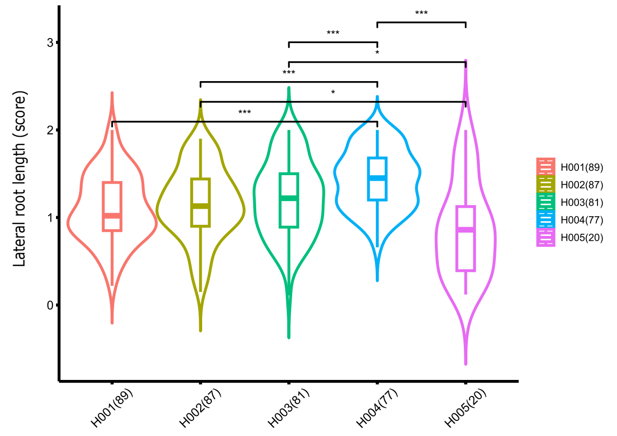

	481975631	481975696	481975827	481976013	481976167	481976200	481976230	freq
		*	*	*	*	*	*	
ALLELE	G/A	C/T	T/C	T/C	C/T	T/C	C/T	
H001	G	T	T	T	C	T	C	89
H002	G	C	T	T	C	T	C	87
H003	G	C	C	T	C	C	C	81
H004	G	C	C	C	C	C	C	77
H005	G	C	T	T	C	C	C	20

HORVU.MOREX.r2.2HG0100950 (qRGA-2H.2)

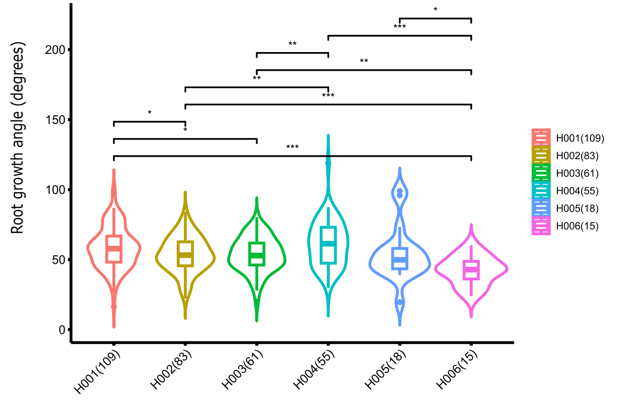

	81639950	81640364	81640367	81640630	81640631	81640640	81640801	81640896	81641008	81641084	81641232	81641253	81641275	81641333	freq
	*	*			*	*		*		*	*	*		*	
ALLELE	T/G	T/G	T/C	C/T	C/G	C/T	G/A	C/T	C/T	G/A	G/A	G/T	G/A	G/C	
H001	G	T	T	C	C	C	A	C	C	G	G	G	G	G	109
H002	T	T	T	C	G	C	G	C	C	G	G	G	G	G	83
H003	T	T	T	C	C	C	G	C	C	G	G	G	G	G	61
H004	T	T	T	C	C	C	G	C	C	G	A	G	G	G	55
H005	T	T	C	C	C	C	A	T	C	G	G	G	G	G	18
H006	T	G	T	C	C	C	G	C	T	G	G	G	G	C	15

HORVU.MOREX.r2.6HG0503680 (qRGA-6H.2)

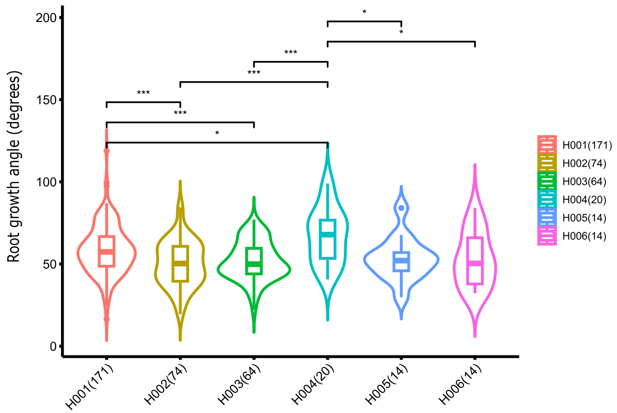

	472581996	472582038	472583171	472583385	472583450	472583544	472583661	472583685	472583689	472583712	472583946	472583955	472583991	472584045	freq
					*				*	*					
ALLELE	C/T	C/T	C/T	A/G	C/T	T/A	C/T	C/T	A/T	C/G	C/T	C/T	G/A	G/A	
H001	C	C	C	A	C	T	C	C	A	C	C	C	A	G	171
H002	C	C	C	A	C	T	C	C	A	C	T	C	G	G	74
H003	C	C	C	A	C	T	T	C	A	C	C	C	G	G	64
H004	T	T	C	A	C	T	C	C	T	C	C	C	G	A	20
H005	C	C	C	A	C	A	C	C	A	C	C	C	G	G	14
H006	C	C	C	A	C	T	C	C	A	C	C	C	G	G	14

HORVU.MOREX.r2.4HG0278070 (qSRN-4H.1)

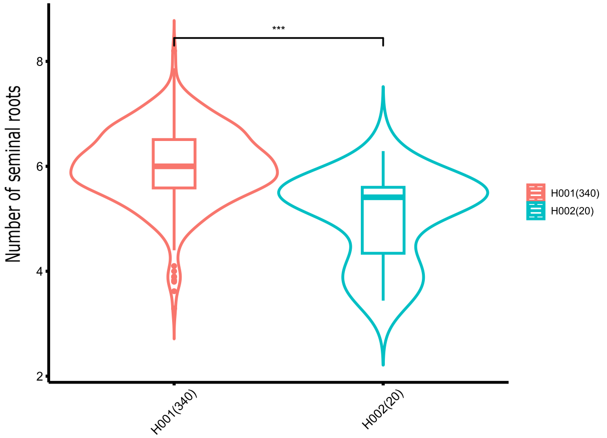

	6234191	6234202	6235699	6235709	6235772	freq
			*	*		
ALLELE	T/C	A/G	C/G	G/A	C/A	
H001	T	A	C	G	C	340
H002	T	A	C	A	A	20

HORVU.MOREX.r2.4HG0284880 (qSRN-4H.2)

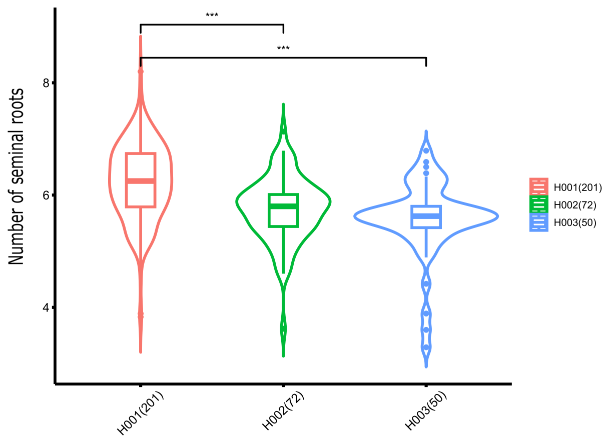

	35432944	35432945	35432949	35432973	35432991	35433223	35433267	35433308	35433653	35433662	35433705	35433722	35433737	35433794	freq
		*				*		*	*	*		*	*	*	
ALLELE	C/A	G/A	G/C	G/A	C/G	G/A	G/C	C/T	G/C	T/C	A/G	A/G	C/A	T/G	
H001	C	G	G	G	C	G	G	C	G	T	A	A	C	T	201
H002	C	G	G	G	C	A	G	T	C	C	G	G	C	G	72
H003	A	A	C	A	G	A	C	C	C	C	G	G	C	G	50

HORVU.MOREX.r2.5HG0433960 (qSRN-5H.2)

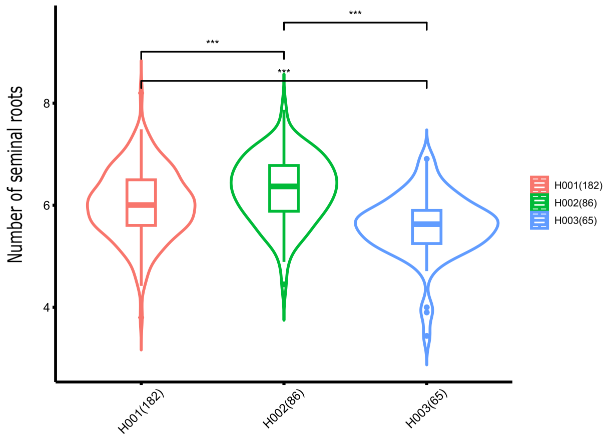

	563448309	563450061	563450065	563450086	563450103	563450115	563450163	563450288	563450385	563450388	563450400	563450440	563450509	563450590	563451440	563451589	563452451	563452480	563452540	563452732	563452897	563452946	563452970	563453010	563453033	freq
	*		*	*				*				*		*	*	*					*			*		
ALLELE	T/C	A/G	C/G	A/C	T/G	A/G	T/C	C/T	T/C	C/T	A/G	G/A	T/C	A/G	A/G	C/G	C/T	G/C	A/C	T/C	C/T	T/C	G/A	G/A	G/A	
H001	C	A	C	A	T	G	T	C	T	C	A	G	T	A	A	C	T	G	A	C	C	C	G	G	G	182
H002	T	A	C	A	T	A	T	C	T	C	A	G	T	A	A	C	C	G	A	T	C	T	G	G	G	86
H003	C	A	C	A	T	A	T	C	T	C	A	G	T	A	A	C	C	G	A	T	C	C	G	G	G	65

HORVU.MOREX.r2.6HG0477060 (qSRN-6H.3)

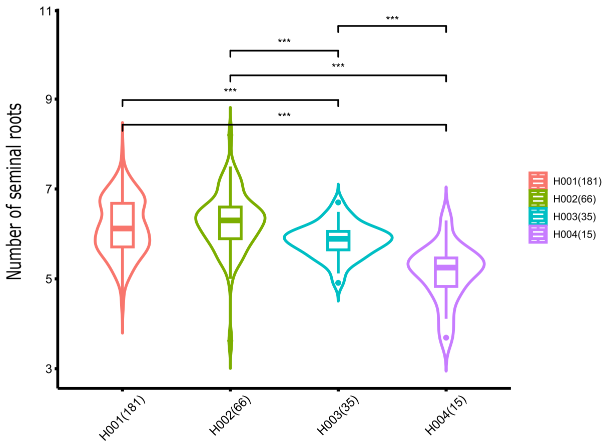

	167812309	167812312	167812332	167812535	167812564	167812584	167812608	167812664	167812693	167812818	167812822	167812843	167812924	167813015	167813030	167813041	167813190	167813211	167813263	167813279	167813408	167813436	167813557	167813573	167813612	167813803	167815542	167815693	167815770	167815860	167815907	167816104	167816381	167816399	167816514	167816957	freq
			*	*			*	*	*				*			*			*	*	*	*		*	*	*		*				*			*	*	
ALLELE	T/C	A/T	G/A	A/G	G/A	T/G	C/T	G/A	G/C	G/A	G/A	T/A	C/A	C/T	A/G	C/G	G/A	G/A	G/A	C/T	G/A	T/A	C/T	G/A	C/T	G/A	T/C	A/G	G/A	C/G	G/T	G/A	A/G	T/C	C/T	T/A	
H001	T	A	G	A	G	T	C	G	G	G	G	T	C	C	A	C	G	G	G	C	G	T	C	G	C	G	T	A	G	C	G	G	A	T	C	T	181
H002	T	A	G	A	G	T	C	G	G	G	G	A	C	C	A	C	G	G	G	C	G	T	C	G	C	G	T	G	G	C	G	G	A	T	C	T	66
H003	T	A	G	A	G	T	C	G	G	G	G	A	C	T	A	C	G	G	G	C	G	T	C	G	C	G	T	G	G	C	G	G	A	T	C	T	35
H004	T	A	G	G	A	G	C	G	G	G	A	A	C	C	A	C	G	A	G	T	A	T	C	G	C	G	T	G	G	C	G	G	G	C	T	T	15
