# Supplementary material for: Genetic dissection of root traits in barley identifies major QTLs and domestication signature
Source: Plant Cell Rep. 2026 May 23;45(6):174. doi: 10.1007/s00299-026-03852-3 (PMC13198497; doi:10.1007/s00299-026-03852-3)
Supplement: Supplementary file 4 — Supplementary file4 (DOCX 52 KB) [file 299_2026_3852_MOESM4_ESM.docx]

**Supplementary text 1**

**Candidate genes for Average root length (ARL), Lateral root length (LRL) and Lateral root density (LRD)**

As regard ARL, at *qARL-1H.1*, two genes were identified as major candidate genes, *HORVU.MOREX.r2.1HG0000900 and* *HORVU.MOREX.r2.1HG0000910*. Haplotypes #4 and #5 of *HORVU.MOREX.r2.1HG0000900* showed highest ARL values and contained only one unique common missense SNP (at chr. position 1H:2224685) causing Q28K amino acid substitution (Fig. S3). *HORVU.MOREX.r2.1HG0000900* encodes a pectate lyase protein, known to play an important role in cell wall formation (Freshour et al. 1996; Somssich et al. 2016). Pectate lyase participates in the degradation of cell wall pectin and its overexpression promotes the elongation of several organs including primary root in *Arabidopsis* (Sun et al. 2020). At the same QTL, haplotypes #3 and #7 of *HORVU.MOREX.r2.1HG0000910* gene had significantly higher ARL value than other haplotypes, with two SNPs at chr. positions 1H:2239529 and 1H:2239529, causing G19R and S18P amino acid substitutions. *HORVU.MOREX.r2.1HG0000910* encodes a jasmonate induced protein. In rice, jasmonate signaling suppresses crown root and primary root elongation in a manner dependent on the F-box protein CORONATINE INSENSITIVE1 (COI1). Mutants in specific JA receptors (e.g., *OsCOI2*) show altered sensitivity to JA, confirming that JA perception itself contributes to reduced root length (Howe et al. 2018; Li et al. 2022; Han et al. 2023; Nguyen et al. 2023).

At *qARL-5H.2*, barley accessions carrying *HORVU.MOREX.r2.5HG0439520* haplotypes #5 and #6 exhibited high ARL values (Fig. S3). These haplotypes contain multiple missense mutations resulting in amino acid changes affecting protein sequence. *HORVU.MOREX.r2.5HG0439520* encodes an F-box protein. F-box proteins have been repeatedly identified as involved in root growth, both as indirect regulators of the stress response and as directly involved in the regulation of cell division independent of stress conditions (Dong et al. 2006; Yan et al. 2011; Zheng et al. 2011; Abd-Hamid et al. 2020).

Taking into consideration LRL, a major QTL, *qLRL_5H.2,* co-maps with *MQTL9-5* identified for different root traits in a syntenic region in rice (Daryani et al. 2022). *qLRL_5H.2* includes the *HORVU.MOREX.r2.5HG0405780* gene. Haplotype #4 is characterized by one unique SNPs (5H:481976013) resulting in K416R amino acid substitutions. This gene encodes a member of WPP-domain proteins, which are associated with nuclear envelope in Arabidopsis. RNA interference-based suppression of the Arabidopsis WPP family caused shorter primary roots and a reduced number of lateral roots, through the reduction of the mitotic activity (Patel et al. 2004; Du et al. 2022). WIPs, a family of WPP-domain interacting proteins, may modulate auxin responses and influence lateral root density and growth across diverse plant species (Crawford et al. 2015; Roldan et al. 2020; Dwivedi et al. 2024).

As regards LRD, *qLRD_2H.3* co-maps also with a QTL identified in rice for root traits *MQTL2-4* (Daryani et al. 2022). It contains the putative candidate gene *HORVU.MOREX.r2.2HG0151020*. Haplotype 3# exhibited a unique SNP (2H:572931283) leading to an amino acid substitution G128D. This gene is expressed at a relatively high level in the barley root elongation zone and encodes a protein with a domain of unknown function, DUF761. Evidence observed in Arabidopsis plants overexpressing the DUF761 domain suggests a role for DUF761-containing proteins in altered root morphology, specifically affecting primary root elongation (Zhang et al. 2019).

**References**

Abd-Hamid N-A, Ahmad-Fauzi M-I, Zainal Z, Ismail I (2020) Diverse and dynamic roles of F-box proteins in plant biology. Planta 251:68

Crawford BCW, Sewell J, Golembeski G, et al (2015) Plant development. Genetic control of distal stem cell fate within root and embryonic meristems. Science 347:655–659

Daryani P, Darzi Ramandi H, Dezhsetan S, et al (2022) Pinpointing genomic regions associated with root system architecture in rice through an integrative meta-analysis approach. Züchter Genet Breed Res 135:81–106

Dong L, Wang L, Zhang Y, et al (2006) An auxin-inducible F-box protein CEGENDUO negatively regulates auxin-mediated lateral root formation in Arabidopsis. Plant Mol Biol 60:599–615

Du Y, Roldan MVG, Haraghi A, et al (2022) Spatially expressed WIP genes control Arabidopsis embryonic root development. Nat Plants 8:635–645

Dwivedi V, Pal L, Singh S, et al (2024) The chickpea WIP2 gene underlying a major QTL contributes to lateral root development. J Exp Bot 75:642–657

Freshour G, Clay RP, Fuller MS, et al (1996) Developmental and tissue-specific structural alterations of the cell-wall polysaccharides of Arabidopsis thaliana roots. Plant Physiol 110:1413–1429

Han X, Kui M, He K, et al (2023) Jasmonate-regulated root growth inhibition and root hair elongation. J Exp Bot 74:1176–1185

Howe GA, Major IT, Koo AJ (2018) Modularity in jasmonate signaling for multistress resilience. Annu Rev Plant Biol 69:387–415

Li M, Zhu Y, Li S, et al (2022) Regulation of phytohormones on the growth and development of plant root hair. Front Plant Sci 13:865302

Nguyen HT, Cheaib M, Fournel M, et al (2023) Genetic analysis of the rice jasmonate receptors reveals specialized functions for OsCOI2. PLoS One 18:e0291385

Patel S, Rose A, Meulia T, et al (2004) Arabidopsis WPP-domain proteins are developmentally associated with the nuclear envelope and promote cell division. Plant Cell 16:3260–3273

Roldan MVG, Izhaq F, Verdenaud M, et al (2020) Integrative genome-wide analysis reveals the role of WIP proteins in inhibition of growth and development. Commun Biol 3:239

Somssich M, Khan GA, Persson S (2016) Cell wall heterogeneity in root development of Arabidopsis. Front Plant Sci 7:1242

Sun H, Hao P, Gu L, et al (2020) Pectate lyase-like Gene GhPEL76 regulates organ elongation in Arabidopsis and fiber elongation in cotton. Plant Sci 293:110395

Yan Y-S, Chen X-Y, Yang K, et al (2011) Overexpression of an F-box protein gene reduces abiotic stress tolerance and promotes root growth in rice. Mol Plant 4:190–197

Zhang Y, Zhang F, Huang X (2019) Characterization of an Arabidopsis thaliana DUF761-containing protein with a potential role in development and defense responses. Theor Exp Plant Physiol 31:303–316

Zheng X, Miller ND, Lewis DR, et al (2011) AUXIN UP-REGULATED F-BOX PROTEIN1 regulates the cross talk between auxin transport and cytokinin signaling during plant root growth. Plant Physiol 156:1878–1893
